# A systematic efficacy analysis of tuberculosis treatment with BPaL‐containing regimens using a multiscale modeling approach

**DOI:** 10.1002/psp4.13117

**Published:** 2024-02-26

**Authors:** Maral Budak, Laura E. Via, Danielle M. Weiner, Clifton E. Barry, Pariksheet Nanda, Gabrielle Michael, Khisimuzi Mdluli, Denise Kirschner

**Affiliations:** ^1^ Department of Microbiology and Immunology University of Michigan Medical School Ann Arbor Michigan USA; ^2^ Tuberculosis Research Section, Laboratory of Clinical Immunology and Microbiology National Institute of Allergy and Infectious Diseases (NIAID) Bethesda Maryland USA; ^3^ Tuberculosis Imaging Program, Division of Intramural Research NIAID Bethesda Maryland USA; ^4^ Centre for Infectious Diseases Research in Africa Institute of Infectious Disease and Molecular Medicine Observatory Republic of South Africa; ^5^ Department of Medicine University of Cape Town Observatory Republic of South Africa; ^6^ Molecular, Cellular and Developmental Biology University of Michigan Ann Arbor Michigan USA; ^7^ Bill & Melinda Gates Medical Research Institute Cambridge Massachusetts USA

## Abstract

Tuberculosis (TB) is a life‐threatening infectious disease. The standard treatment is up to 90% effective; however, it requires the administration of four antibiotics (isoniazid, rifampicin, pyrazinamide, and ethambutol [HRZE]) over long time periods. This harsh treatment process causes adherence issues for patients because of the long treatment times and a myriad of adverse effects. Therefore, the World Health Organization has focused goals of shortening standard treatment regimens for TB in their End TB Strategy efforts, which aim to reduce TB‐related deaths by 95% by 2035. For this purpose, many novel and promising combination antibiotics are being explored that have recently been discovered, such as the bedaquiline, pretomanid, and linezolid (BPaL) regimen. As a result, testing the number of possible combinations with all possible novel regimens is beyond the limit of experimental resources. In this study, we present a unique framework that uses a primate granuloma modeling approach to screen many combination regimens that are currently under clinical and experimental exploration and assesses their efficacies to inform future studies. We tested well‐studied regimens such as HRZE and BPaL to evaluate the validity and accuracy of our framework. We also simulated additional promising combination regimens that have not been sufficiently studied clinically or experimentally, and we provide a pipeline for regimen ranking based on their efficacies in granulomas. Furthermore, we showed a correlation between simulation rankings and new marmoset data rankings, providing evidence for the credibility of our framework. This framework can be adapted to any TB regimen and can rank any number of single or combination regimens.

**Study Highlights**

**WHAT IS THE CURRENT KNOWLEDGE ON THE TOPIC?**

Tuberculosis (TB) kills 1.5 million people every year. To reduce the deaths caused by TB, many promising antibiotics, including the bedaquiline, pretomanid, and linezolid (BPaL) regimen, have been discovered that would potentially outperform the current standard treatment. Because combination treatment is necessary for TB, finding the best combination is an expensive process as a result of the high number of possible combinations.

**WHAT QUESTION DID THIS STUDY ADDRESS?**

How can we assess the efficacies of various combination treatments for TB containing BPaL in a more efficient way to discover a better combination than the standard treatment?

**WHAT DOES THIS STUDY ADD TO OUR KNOWLEDGE?**

We developed an agent‐based pharmacokinetic/pharmacodynamic model of tuberculosis to predict efficacies of various regimens. With a novel ranking method, we are able to rank all new and existing regimens, which correlate well with nonhuman primate studies.

**HOW MIGHT THIS CHANGE DRUG DISCOVERY, DEVELOPMENT, AND/OR THERAPEUTICS?**

Our method is vital to help narrow the design space of possible drug regimens for TB, to screen various regimen combinations in a highly efficient way and to predict their efficacies. We can inform both preclinical trials and experiments with our reliable predictions so that more informed and primate‐centric regimen decisions can be made.

## INTRODUCTION

Tuberculosis (TB) is an ancient disease and remains one of the deadliest infectious diseases in the world, having caused 10.6 million new cases and 1.6 million deaths globally in 2021.^1^ Infection is caused by the inhalation of *Mycobacterium tuberculosis* (Mtb). Although progress has been made to understand the immunology of Mtb infection since its discovery in 1882, much remains to be learned to end the global TB epidemic, which is a key goal of the World Health Organization (the End TB Strategy).^2^ To achieve this goal, effort is focused on reducing both the number of TB deaths and the TB incidence rate as well as improving drug treatment and preventive treatment strategies (e.g., vaccines).

Pulmonary Mtb infection leads to the development of multiple lung granulomas, which are hallmark structures, that is, complex lesions composed of a myriad of immune cells, bacteria, and dead tissue. These structures immunologically restrain the bacteria; however, they also provide an isolated, protected, and nutritious niche for Mtb survival.^3^, ^4^ Because of the heterogeneous structure of granulomas and Mtb phenotypes, and the risk of the emergence of drug resistance, combination drug therapy administered for long periods is needed to cure TB.^5^ The current standard TB treatment against drug‐susceptible TB is up to 90% effective when patients are adherent,^6^ with an overall cure rate of up to 85%.^1^ However, it requires the administration of four drugs, isoniazid, rifampicin, pyrazinamide, and ethambutol (HRZE), for long time periods with adverse effects, making adherence challenging.^7^ Toward improving adherence and achieving the final goal of eradicating TB, the World Health Organization's End TB Strategy aims to shorten the TB treatment window by identifying improved regimens that are both more effective and patient friendly.^2^ To that end, new and repurposed potent antibiotics have been studied in combination *in vitro*, *in vivo*, and clinically during the past few decades, such as moxifloxacin,^8^, ^9^, ^10^, ^11^ bedaquiline,^12^, ^13^, ^14^, ^15^ pretomanid,^12^, ^14^, ^16^ linezolid,^13^, ^17^ rifapentine,^18^, ^19^ sutezolid,^20^, ^21^, ^22^ delamanid,^23^, ^24^ OPC‐167832,^23^, ^25^, ^26^ and so on. Recent research efforts are focused on testing various combinations of these antibiotics in patients and treating drug‐susceptible and drug‐resistant disease.^8^, ^12^, ^13^, ^14^, ^15^, ^18^, ^19^, ^23^, ^27^, ^28^, ^29^, ^30^, ^31^, ^32^

The large number of novel and potent antibiotics against Mtb gives rise to excessive numbers of possible drug combination regimens, on the order of 10^17^.^33^ Although preclinical studies with animal models (e.g., nonhuman primates, mice, rabbits) and clinical trials are highly informative, it is not feasible to test each regimen clinically or on animals, as each clinical trial takes at least a few years and costs an average of $40 million,^34^ and each animal study also requires significant resources. *In vitro* experiments are helpful to measure drug activities systematically; however, they lack the immunological effects that may significantly change the course of the treatment. Therefore, an initial screening that is both accurate and efficient is urgently needed to inform preclinical trials and reduce both the cost and time of regimen discovery. For these reasons, we previously developed a computational model that simulates primate granuloma formation resulting from Mtb infection.^35^, ^36^ We are able to use this model to explore pharmacokinetic (PK) and pharmacodynamic (PD) behaviors and efficacies of all drugs within our study to explore antibiotic regimens of Mtb granulomas in an efficient and fast way.^37^, ^38^, ^39^, ^40^ We provided evidence that our model predictions reflect both clinical and *in vivo* predictions for TB treatment with various combinations of isoniazid, rifampicin, pyrazinamide, ethambutol, and moxifloxacin (HRZEM).^11^, ^41^

In this study, we aimed to assess and compare regimen potencies through modeling that will yield more efficient insights into regimen behavior and to complement experimental methods. Here, we extended our previous work on HRZEM^11^, ^41^ and considered three additional drugs that are shown in combination to be effective: bedaquiline, pretomanid, and linezolid. We focus our analyses on the following regimens: bedaquiline, pretomanid, and linezolid (BPaL); HRZE; bedaquiline and pretomanid (BPa); pretomanid, moxifloxacin, and pyrazinamide (PaMZ); bedaquiline, pretomanid, moxifloxacin, and pyrazinamide (BPaMZ); bedaquiline, pretomanid, linezolid, and moxifloxacin (BPaLM); bedaquiline, pretomanid, and pyrazinamide (BPaZ); bedaquiline, pretomanid, and moxifloxacin (BPaM); bedaquiline, pretomanid, linezolid, and pyrazinamide (BPaLZ); isoniazid, rifampicin, pyrazinamide, and moxifloxacin (HRZM); and rifampicin, moxifloxacin, pyrazinamide, and ethambutol (RMZE). This regimen set included regimens that are both very well studied, such as HRZE and BPaL,^13^, ^15^, ^17^, ^42^ and regimens that are efficient based on preliminary studies but currently have insufficient clinical evidence, such as BPaZ,^12^, ^30^ BPaM,^43^ PaMZ,^16^, ^27^, ^31^ BPaMZ,^30^ BPaLM,^28^, ^32^ and BPaLZ.^42^ A unique feature of our framework is that we can “treat” the same exact granulomas with different regimens, thus accurately comparing and ranking them. Thus, we aimed to both assure the validity of our model with the well‐known regimens and assess potencies of additional regimens using our novel approach. Finally, we developed a quantitative pipeline to prioritize and rank regimens based on their rates of granuloma sterilization. We compared our predictions with data sets from marmoset studies, showing correlations between marmoset studies and *GranSim* rankings. Our pipeline has been established with these sets of regimens but can explore any antibiotic combination regimen for TB.

## METHODS

### Simulating antibiotic regimens using *GranSim*

We used *GranSim* (see Methods in Appendix S1 for more details) and simulated each regimen on 1000 granulomas from our five sets of *in silico* granuloma libraries, each set containing 200 granulomas: 100 low–colony forming unit (CFU) and 100 high‐CFU granulomas (Figure S3; see Methods/*in silico* granuloma library section in Appendix S1 for details on how granulomas were categorized).

For the ranking study, we simulated the following regimens for 180 days: HRZE, HRZM, RMZE, BPaL, BPaM, BPaMZ, BPaLZ, PaMZ, BPaLM, BPaZ, and BPa. In addition, we simulated a regimen (labeled as BPaL‐1moL in results), where we dosed BPaL for 30 days and BPa for the rest of the simulation (150 days) to assess the effect of reducing linezolid from BPaL, following the protocols performed in previous studies.^13^, ^17^ We administered each regimen daily with the following doses for individual antibiotics determined by human doses^7^ or human equivalent rabbit doses: 5 mg/kg of isoniazid, 10 mg/kg of rifampicin, 7 mg/kg of moxifloxacin, 20 mg/kg of ethambutol, 25 mg/kg of pyrazinamide, 20 mg/kg of bedaquiline, 20 mg/kg of pretomanid, and 90 mg/kg of linezolid.

To analyze the correlation between marmoset studies and *GranSim* results, we simulated regimens that were performed on marmosets and were treated for 60 days. We executed the matched treatment regimens on the same *in silico* granuloma sets: bedaquiline, pretomanid, isoniazid, pyrazinamide, rifampicin, moxifloxacin, BPa, pretomanid and linezolid (PaL), bedaquiline and linezolid (BL), isoniazid and pyrazinamide (HZ), rifampicin and pyrazinamide (RZ), rifampicin and moxifloxacin (RM), BPaL, HRZE, and RMZE.

### Measurements to assess regimen efficacies and rankings

#### Sterilization time

**FIGURE 1:**
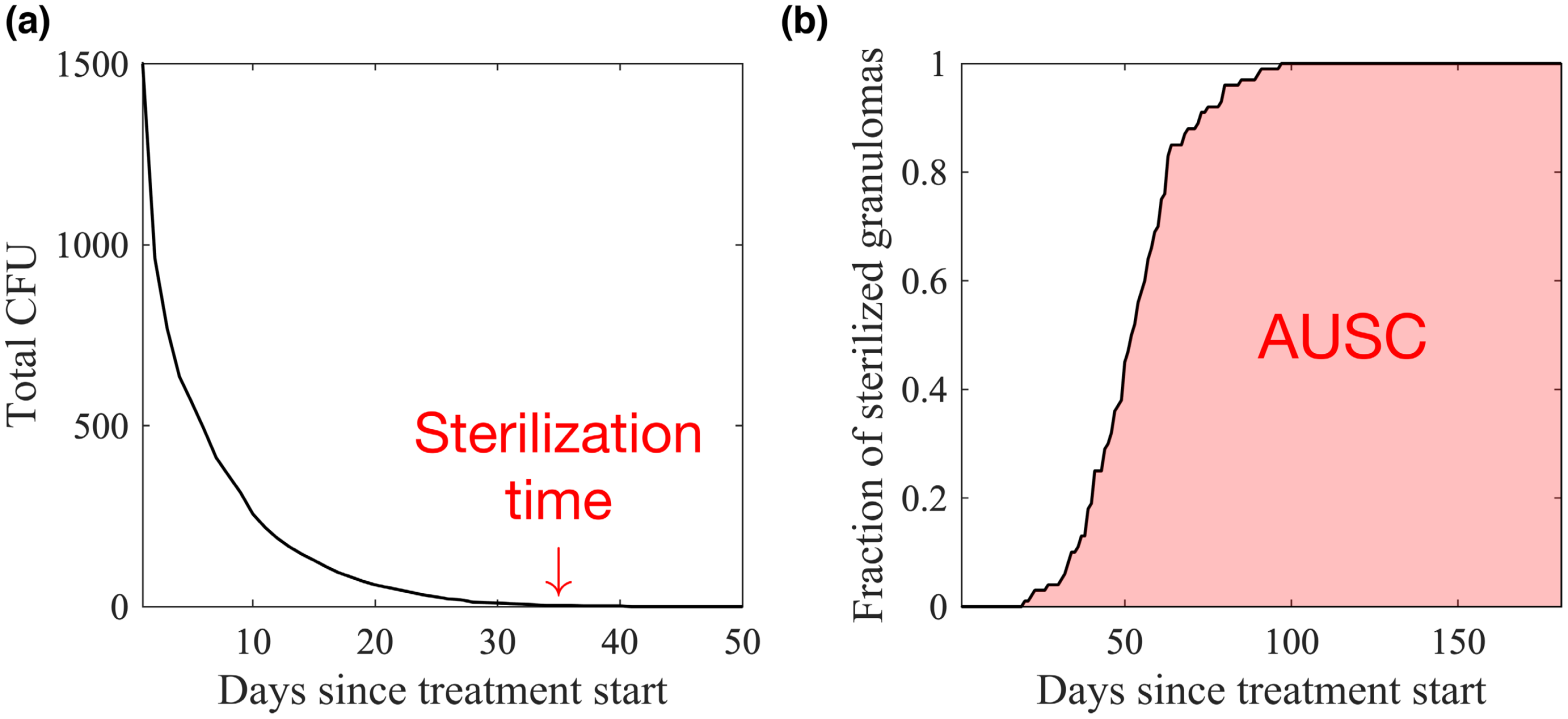
Measurements to assess regimen efficacies: examples of sterilization and area under the curve. Illustrations of (a) sterilization time of a granuloma and (b) area under the sterilization curve (AUSC) for a set of granulomas. (a) Sterilization time for each granuloma is measured by the time needed to administer a treatment to a granuloma until all colony forming units (CFUs) within that granuloma are killed. (b) Once the sterilization time of each granuloma in a granuloma set is calculated, we plot the fraction of granulomas with zero CFU (i.e., sterilized granulomas) in the granuloma set with respect to the treatment time. We consider treatments more effective if the AUSC is higher, which means that granulomas are sterilized more quickly.

We defined the sterilization time of each granuloma as the treatment time required for each granuloma to clear all CFUs (Figure 1a). Lower values of sterilization time represented faster sterilization, hence better regimen efficacy. A key difference between modeling and animal studies is that we can identify with certainty that a granuloma has completely sterilized, whereas *in vivo* that is harder to ensure, which leads to relapsing models of disease, for example, in mice studies.^10^, ^29^

#### Area under the sterilization curve

For each set (five in total) of *in silico* granuloma libraries that contains 200 granulomas each, we determined sterilization times of each granuloma within the set. Then, we plotted the cumulative fraction of sterilized granulomas each day (Figure 1b). We measured the area under the sterilization curve (AUSC) to assess the efficacy of each treatment regimen (Figure 1b). The AUSC values were determined based on the sterilization curve between 0 and 180 days. Higher AUSC values represented faster sterilization, which imply more effective regimens. Note that the toxicities of drugs are not considered.

#### Calculation of a novel score for ranking regimens in a regimen set

A *regimen set* is a group of regimens to be ranked. In this study, we formed regimen sets based on the regimens we intend to rank (e.g., clinically relevant regimens, regimens in marmoset studies). To rank regimens in the regimen set, we created a novel score for each regimen. Calculating a score for a regimen (reference regimen) involved a pairwise, one‐tailed *t*‐test between the AUSC of the reference regimen and the AUSCs of the rest of the regimens in the regimen set, assuming the AUSCs of regimens are normally distributed. For example, we considered HRZE as the reference regimen and compared the performance of HRZE with the performance of all the other regimens under study. We considered that the reference regimen performed better (or worse) than the compared regimen if the AUSCs of the reference regimen are significantly higher (or lower) than the AUSCs of the compared regimen (*p* < 0.05, one‐tailed *t*‐test). We grouped the regimens that have significantly higher and lower AUSCs compared with the reference regimen as “more potent” and “less potent” regimens, respectively. Then, we counted the number of more and less potent regimens to calculate a ranking score for the reference regimen as:

(1) $$\begin{array}{c} {\text{Ranking score}}_{\text{reference regimen}}\\ \;=\left(\mathrm{no}.\;\text{of more potent regimens than the reference regimen}\right)\\ \;-\left(\mathrm{no}.\;\text{of less potent regimens than the reference regimen}\right) \end{array}$$

We used this score to rank regimens, such that regimens with higher scores had higher rankings, regimens with lower scores had lower rankings, and regimens with the same score had the same ranking. Note that this ranking method gave us insight into how well each regimen performs overall within a specific regimen set. A regimen in a higher rank did not necessarily mean that it was significantly better than the regimens in the lower ranks. Instead, it meant that a higher ranked regimen outperforms more regimens than lower ranked regimens within that regimen set. This method is more advantageous than simply ranking the regimens based on the median AUSCs as we consider the level of significance of the differences between AUSCs. In addition, we identified a group of regimens that are most efficacious candidates if none of the regimens in a regimen set have significantly higher AUSC scores than them.

## RESULTS

In this study, we developed a methodology to quantify and rank efficacies of TB regimens using our computational model that predicts PK/PD within granulomas for single and combined regimens. We considered the following regimens: HRZM, RMZE, BPaL, BPa, HRZE, BPaZ, BPaLZ, BPaM, BPaMZ, PaMZ, BPaLM, and BPaL‐1moL. Furthermore, we validated our results with *in vivo* nonhuman primate studies to provide a reliable approach of predicting potencies of TB regimens in a granuloma‐centric model.

### Regimen ranking using AUSC measurements

**FIGURE 2:**
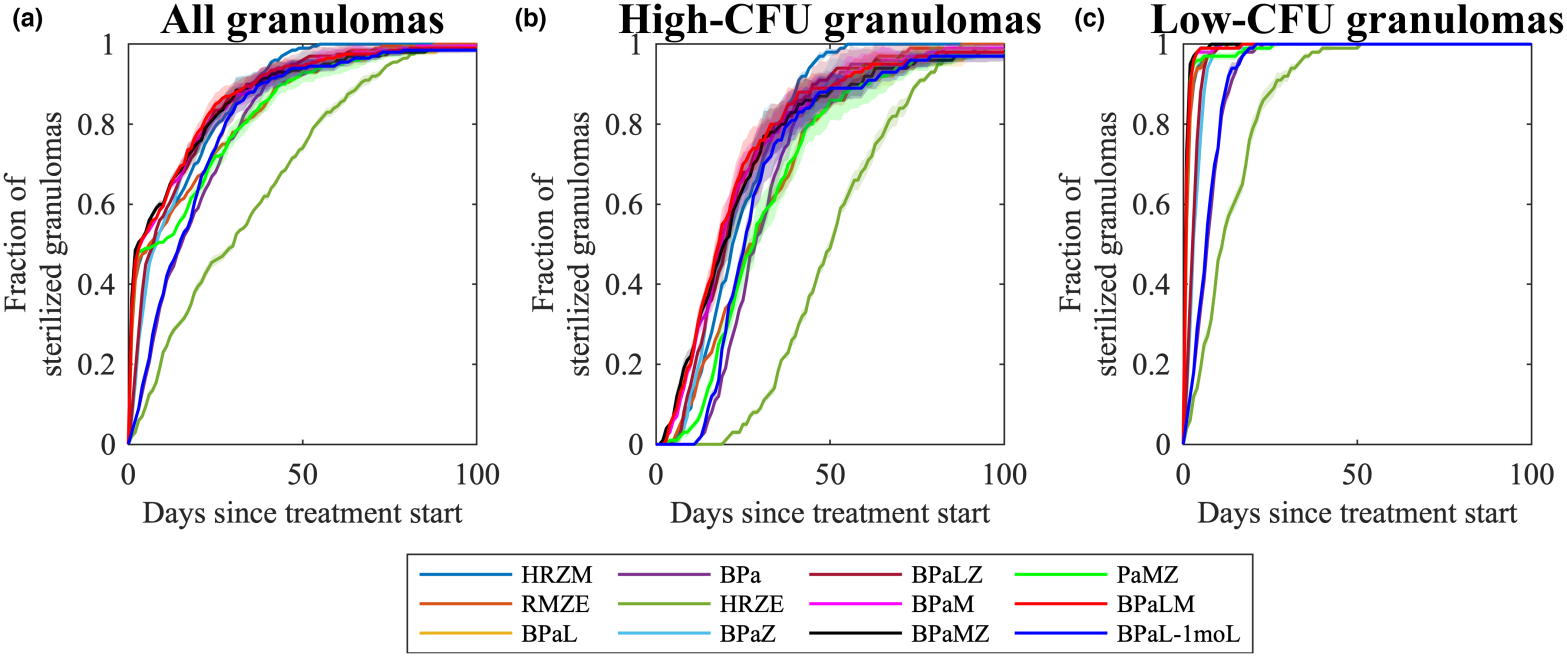
Comparison of simulated regimens with respect to sterilization times. We begin with 1000 granulomas (five groups with 200 granulomas each) from the *in silico* granuloma library. These granulomas were simulated for 300 days after infection. Then we simulated the addition of 12 different antibiotic regimens and report the fraction of sterilized granulomas during the course of treatment for (a) all 1000 granulomas, (b) 500 high‐CFU granulomas, and (c) 500 low‐CFU granulomas. Solid lines represent the median of sterilization fractions of these five groups, and the shaded areas around each line are the relatively small standard error among these five groups. (See Figure S4A for analysis of granuloma‐level variation rather than the group‐level variation shown here.) BPa, bedaquiline and pretomanid; BPaL, bedaquiline, pretomanid, and linezolid; BPaLM, bedaquiline, pretomanid, linezolid, and moxifloxacin; BPaLZ, bedaquiline, pretomanid, linezolid, and pyrazinamide; BPaM, bedaquiline, pretomanid, and moxifloxacin; BPaMZ, bedaquiline, pretomanid, moxifloxacin, and pyrazinamide; BPaZ, bedaquiline, pretomanid, and pyrazinamide; CFU, colony forming unit; HRZE, isoniazid, rifampicin, pyrazinamide, and ethambutol; HRZM, isoniazid, rifampicin, pyrazinamide, and moxifloxacin; PaMZ, pretomanid, moxifloxacin, and pyrazinamide; RMZE, rifampicin, moxifloxacin, pyrazinamide, and ethambutol.

We simulated treatment with each regimen starting at day 300 after infection for 180 days and examined numbers and times of sterilized granulomas. To determine the efficacies of each regimen of interest, we evaluated the fraction of sterilized granulomas at each time step posttreatment considering all granulomas as well as separating out both high‐ and low‐CFU granulomas (Figure 2). We observed that HRZE sterilizes granulomas slower than other regimens in all three CFU groups (Figure 2).

**FIGURE 3:**
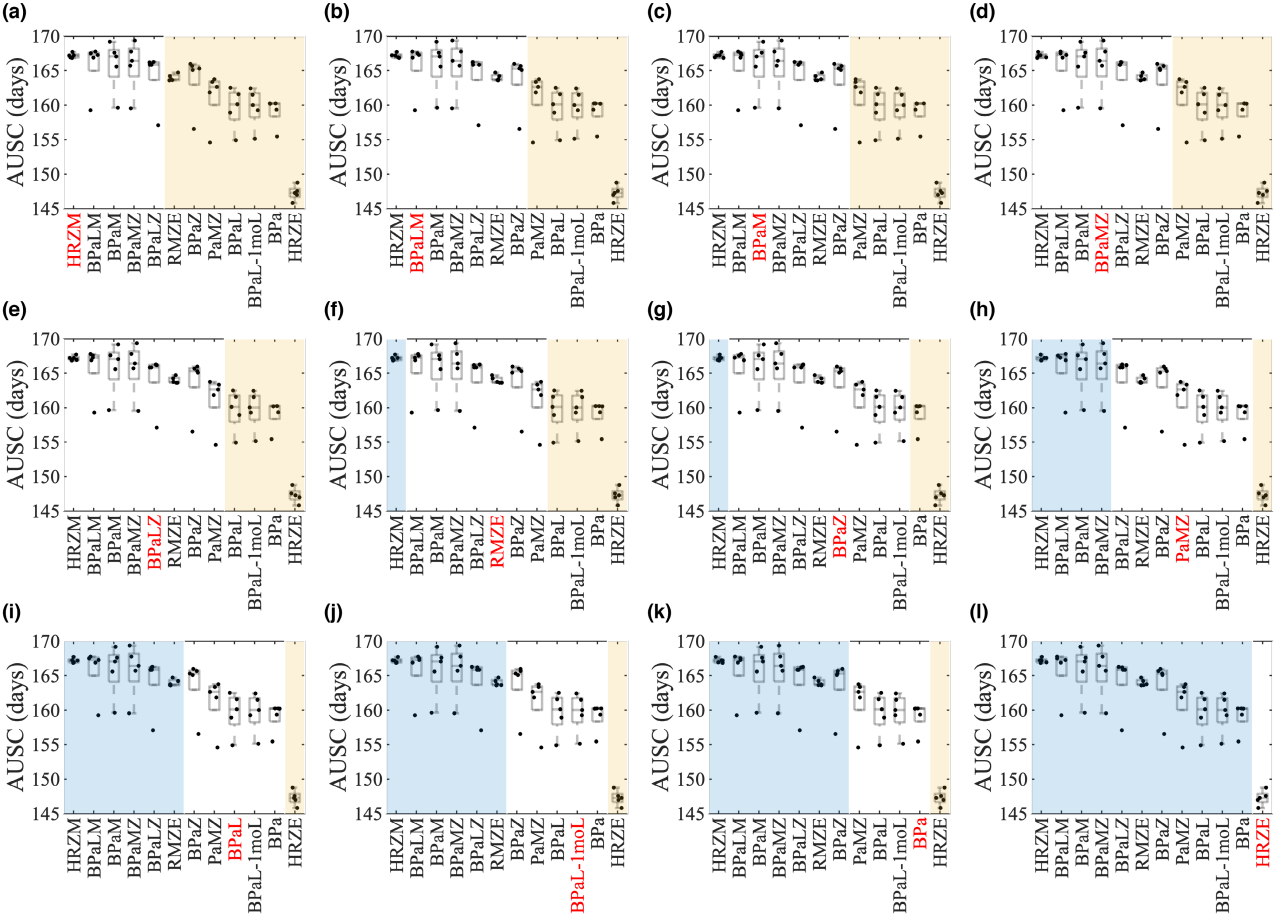
AUSC plots of regimens with all granulomas combined using a novel ranking score. We derived box plots for AUSCs upon treatment for each regimen, considering all 1000 granulomas combined. Each dot represents the AUSC of one group of 200 granulomas. In each panel, the regimen in red is the reference regimen (see the Methods section): (a) HRZM, (b) BPaLM, (c) BPaM, (d) BPaMZ, (e) BPaLZ, (f) RMZE, (g) BPaZ, (h) PaMZ, (i) BPaL, (j) BPaL‐1moL, (k) BPa, (l) HRZE. The reference regimen is compared pairwise to each other regimen (in black). We summarize the outcomes as follows: the regimens in the yellow‐shaded areas indicate that these regimens have significantly lower AUSCs than the reference regimen. Likewise, the regimens in the blue‐shaded areas indicate that these regimens have significantly higher AUSCs than the reference regimen. The difference between the AUSCs of the regimens with a white background and the reference regimen is insignificant (one‐tailed, pairwise *t*‐test, *p* < 0.05). AUSC, area under the sterilization curve; BPa, bedaquiline and pretomanid; BPaL‐1moL, bedaquiline, pretomanid, linezolid for days 1‐30, bedaquiline and pretomanid for days 31‐180; BPaL, bedaquiline, pretomanid, and linezolid; BPaLM, bedaquiline, pretomanid, linezolid, and moxifloxacin; BPaLZ, bedaquiline, pretomanid, linezolid, and pyrazinamide; BPaM, bedaquiline, pretomanid, and moxifloxacin; BPaMZ, bedaquiline, pretomanid, moxifloxacin, and pyrazinamide; BPaZ, bedaquiline, pretomanid, and pyrazinamide; HRZE, isoniazid, rifampicin, pyrazinamide, and ethambutol; HRZM, isoniazid, rifampicin, pyrazinamide, and moxifloxacin; PaMZ, pretomanid, moxifloxacin, and pyrazinamide; RMZE, rifampicin, moxifloxacin, pyrazinamide, and ethambutol.

To analyze the efficacies of regimens in a more quantitative way, we evaluated the AUSCs of each regimen for all 1000 (five groups of 200) granulomas (Figure 3) as well as 500 high‐CFU (Figure S5) and 500 low‐CFU granulomas (Figure S6) separately. To rank regimens based on their AUSCs, we calculated a score for each regimen, where the score of a reference regimen depends on the number of regimens having significantly higher (regimens with the blue‐shaded background) or significantly lower (regimens with the yellow‐shaded background) AUSCs than the reference regimen in each case (Table 1, Tables S5–S7) (see the Methods section for more details). As we similarly observed in the sterilization curves (Figure 2), HRZE has the worst ranking in all granuloma groups. However, we showed that higher ranking regimens depended on the type of granuloma: HRZM was one of the best regimens for the high‐CFU granulomas along with BPaM, BPaLM, BPaZ, BPaLZ, and BPaMZ (Table S6), whereas HRZM was not one of the top regimens for the low‐CFU granulomas. BPaMZ, BPaM, and BPaLM worked best for the low‐CFU granulomas as in the high‐CFU granuloma case (Table S7). Although we have ranked them in a specific order, we believe our approach best identifies a group of regimens as top rather than identifying a single top regimen. This is a granuloma scale model that does not account for toxicity, cost, and so on.

**TABLE 1:** Rankings based on scores for each regimen set on the total 1000 simulated granuloma set.

Reference regimen	Rank	No. of regimens significantly worse than the reference regimen (*w*)	No. of regimens significantly better than the reference regimen (*b*)	Score (*w*−*b*)^a^
HRZM	1	7	0	7
BPaLM	2	5	0	5
BPaM	2	5	0	5
BPaMZ	2	5	0	5
BPaLZ	5	4	0	4
RMZE	6	4	1	3
BPaZ	7	2	1	1
PaMZ	8	1	4	−3
BPaL	9	1	6	−5
BPaL‐1moL	9	1	6	−5
BPa	11	1	7	−6
HRZE	12	0	11	−11

*Note*: Shown are both ranking scores and rankings for each regimen in the regimen set compared with others for the total combined granulomas (i.e., high‐CFU and low‐CFU granulomas combined). Individual rankings for regimens by low‐ versus high‐CFU granulomas are shown in Tables S3 and S4. Dark‐shaded regimens are candidates for the most potent regimens, and regimens with the white background are candidates for the least potent regimens. The regimens in the light‐shaded area are those regimens for which areas under the sterilization curve are higher (and lower) than at least one regimen in the regimen set.

Abbreviations: BPa, bedaquiline and pretomanid; BPaL, bedaquiline, pretomanid, and linezolid; BPaLM, bedaquiline, pretomanid, linezolid, and moxifloxacin; BPaL‐1moL, bedaquiline, pretomanid, linezolid for days 1–30, bedaquiline and pretomanid for days 31–180; BPaLZ, bedaquiline, pretomanid, linezolid, and pyrazinamide; BPaM, bedaquiline, pretomanid, and moxifloxacin; BPaMZ, bedaquiline, pretomanid, moxifloxacin, and pyrazinamide; BPaZ, bedaquiline, pretomanid, and pyrazinamide; CFU, colony forming unit; HRZE, isoniazid, rifampicin, pyrazinamide, and ethambutol; HRZM, isoniazid, rifampicin, pyrazinamide, and moxifloxacin; PaMZ, pretomanid, moxifloxacin, and pyrazinamide; RMZE, rifampicin, moxifloxacin, pyrazinamide, and ethambutol.

^a^ Note that the score is the difference of *w* and *b*.

When listing the rankings of all regimens (see the Methods section), we observed that HRZM still had the best ranking, and BPaLM, BPaM, BPAMZ, and BPaLZ are the top regimens for the case where all granulomas are combined (Table 1; see Table S5 for more details). BPa and BPaL performed slightly better than HRZE. Note that they still had lower rankings than the rest of the regimens in the regimen set and that regimens BPaLM and BPaM were ranked in the top three in all granuloma groups.

### Candidates for the most and least potent regimens

**FIGURE 4:**
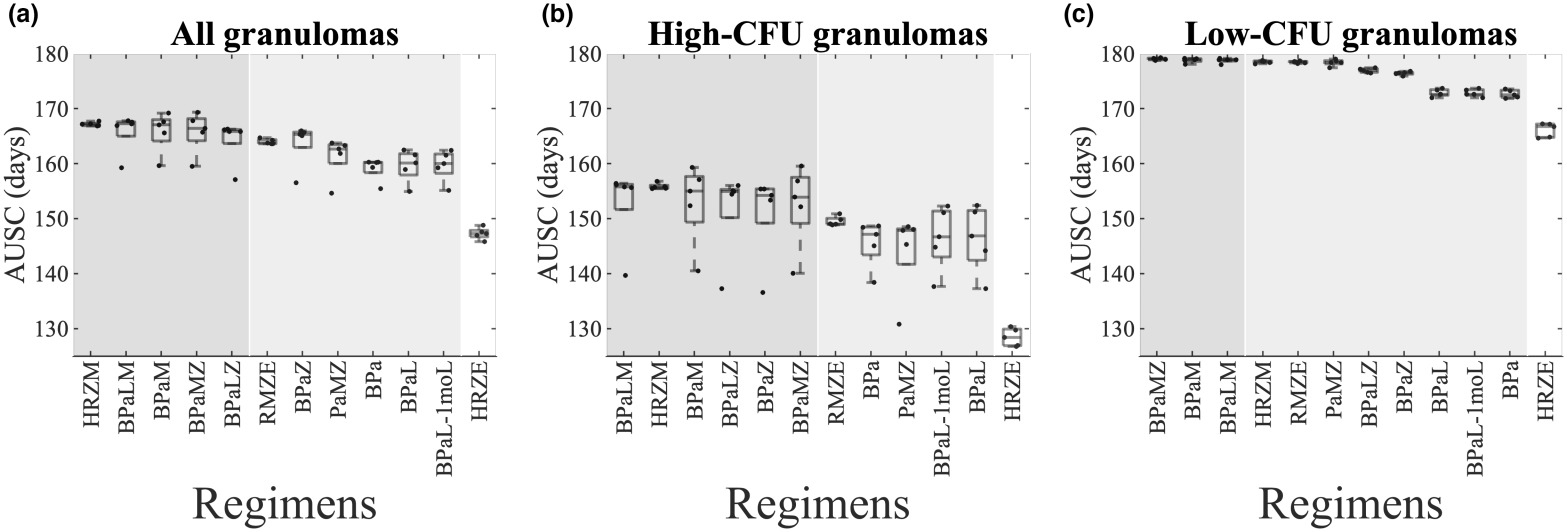
Candidates for the most and least potent regimens. Shown are AUSC box plots with the candidates for the most potent (regimens in the dark–shaded areas) and least potent regimens (regimens with the white background) in the regimen set considering (a) all total 1000 granulomas combined, (b) 500 high‐CFU granulomas, and (c) 500 low‐CFU granulomas. Candidates for the most or least potent regimens are those whose AUSCs are not significantly lower or higher than any other regimen, respectively. The regimens in the light–shaded area are those regimens for which AUSCs are higher (and lower) than at least one regimen in the regimen set. Box plots are ordered based on the medians of regimens' AUSCs. AUSC, area under the sterilization curve; BPa, bedaquiline and pretomanid; BPaL, bedaquiline, pretomanid, and linezolid; BPaLM, bedaquiline, pretomanid, linezolid, and moxifloxacin; BPaLZ, bedaquiline, pretomanid, linezolid, and pyrazinamide; BPaM, bedaquiline, pretomanid, and moxifloxacin; BPaMZ, bedaquiline, pretomanid, moxifloxacin, and pyrazinamide; BPaZ, bedaquiline, pretomanid, and pyrazinamide; CFU, colony forming unit; HRZE, isoniazid, rifampicin, pyrazinamide, and ethambutol; HRZM, isoniazid, rifampicin, pyrazinamide, and moxifloxacin; PaMZ, pretomanid, moxifloxacin, and pyrazinamide; RMZE, rifampicin, moxifloxacin, pyrazinamide, and ethambutol.

Based on our analyses of calculating a ranking score for each regimen (Figure 3, Figures S5 and S6), we identified candidates for the most (high score) and least (low score) potent regimens in the regimen set. We presumed that a reference regimen could potentially be “the best” regimen if no other regimen had significantly higher AUSCs than this reference regimen. This means that there would not be any blue‐shaded region on the panels where the reference regimen was compared with the rest of the regimens in the regimen set. Likewise, we concluded that a reference regimen could potentially be “the worst” regimen if no other regimen had significantly lower AUSCs than this reference regimen. In other words, there would be no yellow‐shaded region on the panels where the reference regimen is compared with the rest of the regimens in the regimen set. We predicted that HRZE was the worst regimen, that is, the least potent regimen, in both individual granuloma sets (high‐CFU and low‐CFU granulomas, respectively) as well as total combined (Figure 4, white region in all panels). We provided evidence that BPaLM, BPaMZ, and BPaM were candidates for the most potent regimen in all cases (Figure 4, dark purple region in all panels). Moreover, HRZM and BPaLZ were candidates for best regimens in high‐CFU and total granulomas (Figure 4a,b), and yet also potent in low‐CFU granulomas (Figure 4c). BPaZ was the most potent regimen candidate for high‐CFU granulomas alone (Figure 4b). However, BPaZ was also efficient in other granuloma groups as well.

### Comparison to marmoset studies

**FIGURE 5:**
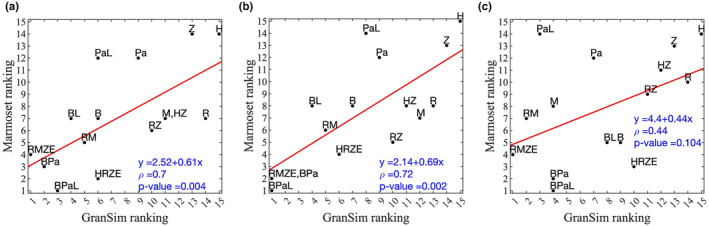
Correlation of *GranSim* rankings to marmoset rankings for all regimens. We ranked regimens using *GranSim* simulations when (a) all granulomas, (b) high–colony forming unit (CFU) granulomas (granulomas with ≥10^4^ CFUs; see Appendix S1), and (c) low‐CFU granulomas (granulomas with <10^4^ CFUs) are treated with regimens for 2 months. We rank regimens using lesion‐scale CFU counts from marmosets treated with regimens for 2 months (a) with all lesion types, (b) with necrotic/caseous lesions, and (c) with fibrotic/normal lesions. (a) We correlate the rankings of *GranSim* with all granulomas to the rankings from marmosets with all lesion types. (b, c) We correlated the rankings of *GranSim* with high‐ and low‐CFU granulomas to the rankings from marmosets with necrotic/caseous and fibrotic/normal lesions, respectively (Spearman's rank correlation). In each panel, the red line is the best linear fit (represented by the equation in the lower right corner, first row of each panel). B, bedaquiline; BL, bedaquiline and linezolid; BPa, bedaquiline and pretomanid; BPaL, bedaquiline, pretomanid, and linezolid; H, isoniazid; HRZE, isoniazid, rifampicin, pyrazinamide, and ethambutol; HZ, isoniazid and pyrazinamide; M, moxifloxacin; Pa, pretomanid; PaL, pretomanid and linezolid; R, rifampicin; RM, rifampicin and moxifloxacin; RZ, rifampicin and pyrazinamide; RMZE, rifampicin, moxifloxacin, pyrazinamide, and ethambutol; Z, pyrazinamide.

To validate the credibility of our ranking method, we compared our results with newly generated *in vivo* marmoset studies (see Methods in Appendix S1 for the details of marmoset experiments). For that purpose, we determined CFU counts for individual lesions from marmosets treated for 2 months with various single and combination regimens: bedaquiline, pretomanid, isoniazid, pyrazinamide, rifampicin, moxifloxacin, BPa, PaL, bedaquiline and linezolid (BL), isoniazid and pyrazinamide (HZ), rifampicin and pyrazinamide (RZ), rifampicin and moxifloxacin (RM), BPaL, HRZE, and RMZE (Figure S7). We ranked the efficacies of these regimens in marmosets by the ranking score described (see the Methods section). We performed the ranking using all lesion types from marmosets (Table S9) as well as two subsets of lesion types: caseous/necrotic lesions and fibrotic/normal lesions (Tables S10 and S11). To compare marmoset rankings to *GranSim*, we simulated the same regimens to treat our granuloma sets (high CFU, low CFU, and combined) for 2 months (Figure S8) and ranked them (Table 2 and Tables S12–S14). We compared the correlations of their rankings to marmoset rankings using Spearman's rank correlation (Figure 5), which in general correlated well when considering all types of granulomas in *GranSim* and marmosets (Figure 5a). We showed that correlations between high‐CFU granulomas from *GranSim* with caseous/necrotic granulomas from marmosets yielded a higher *p* value (0.72) than correlations between low‐CFU granulomas from *GranSim* with fibrotic granulomas from marmosets (0.44) (Figure 5b,c). This was an expected result because marmosets develop progressive TB disease that is likely well represented by high‐CFU granulomas in *GranSim*.

**TABLE 2:** Rankings based on scores for each simulated regimen set.

Reference regimen	Rank	No. of regimens significantly worse than the reference regimen (*w*)	No. of regimens significantly better than the reference regimen (*b*)	Score (*w*−*b*)^a^
RMZE	1	13	0	13
BPa	2	12	0	12
BPaL	3	12	1	11
BL	4	11	3	8
RM	5	10	4	6
HRZE	6	7	5	2
PaL	6	7	5	2
Bedaquiline	6	7	5	2
Pretomanid	9	6	8	−2
RZ	10	5	9	−4
HZ	11	3	10	−7
Moxifloxacin	11	3	10	−7
Pyrazinamide	13	2	12	−10
Rifampicin	14	1	13	−12
Isoniazid	15	0	14	−14

*Note*: Ranking scores and ranks of each regimen from marmoset studies simulated in *GranSim*, considering all granulomas, that is, high‐CFU and low‐CFU granulomas combined. Dark‐shaded regimens are candidates for the most potent regimens, and regimens with the white background are candidates for the least potent regimens. The regimens in the light‐shaded area are those regimens for which areas under the sterilization curve are higher (and lower) than at least one regimen in the regimen set.

Abbreviations: BL, bedaquiline and linezolid; BPa, bedaquiline and pretomanid; BPaL, bedaquiline, pretomanid, and linezolid; CFU, colony forming unit; HRZE, isoniazid, rifampicin, pyrazinamide, and ethambutol; HZ, isoniazid and pyrazinamide; PaL, pretomanid and linezolid; RM, rifampicin and moxifloxacin; RMZE, rifampicin, moxifloxacin, pyrazinamide, and ethambutol; RZ, rifampicin and pyrazinamide.

^a^ Note that the score is the difference of *w* and *b*.

## DISCUSSION

Many clinical trials and *in vivo* studies have focused on various combinations of novel antibiotic regimens to improve the treatment of Mtb infection by shortening treatment time and reducing total drug dose. These studies typically compare results against the standard regimen used for drug‐susceptible TB, namely, HRZE (or isoniazid, rifampicin, and pyrazinamide [HRZ] in murine models). The Nix‐TB study suggested that a novel combination regimen, BPaL, is very promising, although the toxic effects of linezolid result in adverse events with a high incidence.^15^ Other studies have focused on minimizing, or ideally removing, linezolid from BPaL while maintaining the efficacy of BPaL.^13^, ^17^, ^42^ These studies provided evidence that the additional bactericidal effect of linezolid on the combination of bedaquiline and pretomanid is minimal, although linezolid has been shown to decrease relapse rates significantly.^13^, ^17^, ^42^ Clinical evidence also suggested that an initial dosing of linezolid for the first 1–2 months combined with bedaquiline and pretomanid increased regimen efficacy^17^, ^42^ and the combination of bedaquiline and pretomanid alone is not sufficient to exceed the performance of HRZE.^27^ In addition, the combination of moxifloxacin^28^, ^32^ or pyrazinamide (PZA)^42^ with BPaL are both shown to be more efficient than BPaL, thus BPaLM and BPaLZ are potential candidates for treatment‐shortening regimens. Although BPaLZ with the absence of linezolid, that is, BPaZ, is still superior to the standard regimen,^12^, ^30^ BPaLM with the absence of linezolid (BPaM) seems to have a comparable bactericidal activity as the standard regimen based on an *in vivo* study with BALB/c mice.^43^ Still, there is insufficient evidence to conclude that BPaM is not superior to HRZE, and clinical studies need to be conducted.

Another regimen of interest is PaMZ, which has been initially shown to have a higher efficacy than HRZE,^16^, ^27^ but other studies provided evidence that PaMZ is not superior than HRZE.^31^ However, the addition of bedaquiline into the PaMZ regimen (BPaMZ) significantly increases bactericidal and sterilizing activity of the regimen, making it superior to HRZE.^30^ Taken together, these results suggested that there are many unknowns, and conflicting results in the literature and all drug regimens for TB need to be analyzed more systematically.

In our rankings of regimens using our new scoring system, regimens that contain moxifloxacin were shown to be more promising. This partly followed because of the bactericidal assays in caseum mimic^44^ that we used to build our PD model. Those data suggested that moxifloxacin has a strong capacity to kill nonreplicating Mtb, and so it was not surprising that moxifloxacin‐containing regimens did well in *GranSim* simulations, where we treated necrotic granulomas. There is some support for these findings in the literature that suggests that moxifloxacin‐containing regimens such as HRZM, RMZE, and BPaMZ are highly effective as a result of moxifloxacin's strong bactericidal activity.^9^, ^10^, ^29^, ^30^, ^45^ However, there are a few conflicting results from clinical trials. HRZM and RMZE failed to exhibit noninferiority to HRZE in the Rapid Evaluation of Moxifloxacin in Tuberculosis (ReMOXTB) study,^8^ whereas the addition of moxifloxacin into BPaZ, that is, BPaMZ, increases the efficacy of BPaZ significantly in mouse models^29^ as well as in clinical trials.^30^ Therefore, we hypothesized that the effect of moxifloxacin may depend on the accompanying antibiotics within a regimen and would like to investigate this hypothesis in future work.

Our rankings suggested that linezolid did not have a significant additional effect on a regimen. The reason for this is that linezolid is not very potent against nonreplicating Mtb in *ex vivo* bactericidal assays that we used to calibrate our PD parameters (Sarathy et al.^44^ and Figure S2I). This results in an insignificant effect on caseous granulomas with many nonreplicating Mtb. Likewise, experimental and clinical studies provided evidence that linezolid has minimal additional bactericidal effect or minimal additional ability to convert the positive sputum cultures of TB patients.^13^, ^17^, ^42^ Moreover, we predicted that BPaLM, BPaMZ, and BPaLZ are the best regimen candidates that agree with clinical or experimental outcomes.^28^, ^30^, ^32^, ^42^

Not all of our predictions agreed with clinical trials or had a high correlation with the marmoset outcomes. For example, regimens containing HRZE perform better in marmosets than *GranSim*, and regimens containing BPaL have higher rankings in *GranSim*. These differences may stem from higher dosing of PZA in marmoset studies resulting in approximately 150% of the human equivalent AUSC, which could potentially lead to slightly increased efficacy of HRZE in marmosets. However, some animal studies provided evidence that BPaL outperforms HRZE, which agreed with *GranSim* studies.^17^, ^42^ Moreover, marmosets tend to have more progressive disease, which explains why our correlations are better with high‐CFU granulomas. We would like to emphasize that we only considered granuloma‐level CFU counts from marmosets and assumed that each granuloma develop independently based on the localized immune response, as shown previously.^46^ We have not taken host‐level effects or toxicity into account while ranking marmoset granulomas; therefore, a host‐level analysis on marmoset data to compare with our host‐level model outcomes would be essential as a next step.

It is important to note a few points about our current model. First, we consider only treatment of pulmonary TB, and we do not explore either extrapulmonary or lymph node granuloma involvement. However, considering that most of the adult world has pulmonary TB, our work is a good first step toward improving treatment regimen predictions. Second, there are many important TB drug studies that base success or failure of treatment on the idea of relapse.^10^, ^29^ Even if a drug regimen has been successful in curing a patient or animal initially, later relapse of the disease suggests that the regimen is not effective in the long term. Granuloma‐scale models like *GranSim* cannot predict whether the disease will relapse, because, by construction, whole‐host scale sources of relapse (e.g., bacterial reservoir in lymph nodes) cannot be captured in detail.^47^ These dynamics would potentially elongate the treatment, explaining the shorter sterilization times in *GranSim* than what has been observed in clinical practices. Therefore, our model predicts mainly the bactericidal activity of regimens rather than curing ability. Current work in our group is exploring a host‐scale model for TB drug simulations.^48^ Another point is that the PD model in *GranSim* is calibrated to *in vitro* assays, which inherently do not have an immunological effect. To overcome this and to prove the credibility and translatability of our model, we validated our results with *in vivo*/clinical data where available (i.e., marmoset and clinical comparisons in this study). Lastly, our PD model does not consider the inoculum effect, which further contributes to the fast sterilization rates attributed to the heterogeneous distribution of Mtb within granulomas. We are currently working to address this issue in our future studies.

There are many new directions we are currently pursuing for this work. First, we would like to include additional antibiotics that have large potential to be successful, such as OPC‐167832, delamanid, sutezolid, and so on. Various combinations of these antibiotics are currently being investigated clinically, for example, delamanid, bedaquiline, OPC‐167832, and sutezolid (DBOS) as well as pretomanid, bedaquiline, OPC‐167832, and sutezolid (PBOS; ClinicalTrials.gov ID: NCT05971602) and bedaquiline, delamanid, and moxifloxacin (BDM; ClinicalTrials.gov: NCT03959566). Our pipeline can easily include these antibiotics in future studies. Although we can continue to include additional antibiotics to study in this framework, one issue becomes the large number of regimens to screen. Even with computational modeling it becomes difficult to simulate and compare these large numbers of regimens. One other approach we have developed is an optimization pipeline specifically for satisfying multiple goals (objectives) using surrogate‐assisted optimization and Pareto fronts.^11^, ^33^ Performing such optimization will help identify good candidates for treatment out of large numbers of identified and putative regimens. Our current version of our optimization pipeline evaluates regimens based on bactericidal activities only. In our future studies, we aim to incorporate known toxicities of regimens into our optimization objective to also discover regimens with minimized toxicities. One key aspect of our work that remains is to perform these simulations on a whole‐host scale. We have now created a multiscale model, *HostSim*,^49^, ^50^ that allows us to simulate drug treatment at the granuloma, host, and population scales. Recently, our group created a Virtual Cohort Framework, did a prototype antibiotic treatment study using *HostSim*, and reported drug efficacy results over all three physiological scales simultaneously, namely, granuloma, individual host, and population scales.^48^ Our next step is to implement fine‐grained PK/PD dynamics into that host‐scale model to revisit these regimen predictions across scales. *HostSim* will also allow us to address the issue of relapse.

## AUTHOR CONTRIBUTIONS

M.B. and D.K. wrote the manuscript. K.M. and D.K. designed the research. M.B., L.E.V., D.M.W., C.E.B., and G.M. performed the research. M.B., P.N., and G.M. analyzed the data. M.B. and D.K. contributed new reagents/analytical tools.

## FUNDING INFORMATION

This work was funded through the Gates Medical Research Institute (D.K.) and by National Institutes of Health Grant R01 AI50684 (D.K.) and the Center for Data‐Driven Drug Development and Treatment Assessment (DATA; D.K. and M.B.) and in part by the intramural research program of National Institute of Allergy and Infectious Diseases (C.E.B., L.E.V.) and the Bill and Melinda Gates Foundation (OPP1162695 to C.E.B.).

## CONFLICT OF INTEREST STATEMENT

The authors declared no competing interests for this work.

## Supporting information


Appendix S1
