# Supplementary material for: A systematic efficacy analysis of tuberculosis treatment with BPaL‐containing regimens using a multiscale modeling approach
Source: CPT Pharmacometrics Syst Pharmacol. 2024 Feb 26;13(4):673–85. doi: 10.1002/psp4.13117 (PMC11015080; doi:10.1002/psp4.13117)
Supplement: Supplementary file 1 — Appendix S1 [file PSP4-13-673-s001.docx]

Supplementary Information for

**A systematic efficacy analysis of tuberculosis treatment with BPaL-containing regimens using a multi-scale modeling approach**

Maral Budak^1^, Laura E. Via^2,3^, Danielle M. Weiner^2,3^, Clifton E. Barry^2,4^ Pariksheet Nanda^1^, Gabrielle Michael^5^, Khisimuzi Mdluli^6^, Denise Kirschner^1^*

**Methods**

Computational modeling of granuloma formation and drug dynamics

## **Overview:** To develop and apply a computational model to study the effects of drug treatment on pulmonary *Mycobacterium tuberculosis* (Mtb) infection, we focused on the dynamics of antibiotics within lung granulomas. Lung granulomas are the main sites of Mtb during pulmonary TB infection, and thus targeting them for drug treatment is an appropriate focus. Lymph nodes (LNs) have been shown to additionally harbor granulomas in some cases, and current work in our group is exploring their role in outcomes. We utilized our existing model of granuloma formation and function in which we have additionally included a sub-model to capture pharmacokinetics (PK) / pharmacodynamics (PD). We included new antibiotics that we have not previously studied (bedaquiline, pretomanid, and linezolid) by calibrating the PK/PD parameters of each drug individually in the model based on data derived from humans, rabbits, and NHP granulomas. We simulated hundreds of different granulomas creating an *in silico* repository of granulomas covering a spectrum of spatial and temporal granuloma types with colony forming unit (CFU) values over a wide range. We repeatedly treated each granuloma with multiple regimens for comparison. This is a unique feature of our framework—that we can treat the same exact sets of granulomas with different regimens. We then create a ranking pipeline where we quantify the success of a regimen based on the rates of granuloma sterilization.

## *GranSim*

*GranSim* is a computational model that has been continuously curated for 20 years on human and nonhuman primate (NHP) datasets [1, 2]. Briefly, *GranSim* is comprised of multiple types of model structures (hybrid), but the overall structure is an agent-based model (ABM). ABMs are discrete and stochastic models that track individual cells and bacteria as ‘agents’. *GranSim* simulates the immune response against Mtb infection in an area of lung tissue (6mm x 6mm) represented by a 300 x 300 spatial grid, where each grid compartment is 20 µm, the size of the largest immune cell type, the macrophage. Immune cells (macrophages and T cells) and Mtb are tracked individually. Agents in an ABM can move and interact with each other on the lung grid according to immunology-based rules, such as chemokine/cytokine secretion, bactericidal activity against Mtb, activation/deactivation of immune cells, etc. (for a complete description of our rules, see http://malthus.micro.med.umich.edu/GranSim/). Each simulation started with an infected macrophage in the center of the simulation grid that triggered recruitment of immune cells from lung draining lymph nodes through lung vascular sources. The interactions defined by the immunology-based rules lead to the formation of granulomas as an emergent behavior (i.e. we do not script it to simulate a granuloma, yet one emerges via every simulation). We simulated three locations and phenotypes of bacteria within granulomas: intracellular Mtb interior to macrophages, extracellular Mtb (within the granuloma tissue), and nonreplicating Mtb that are trapped within the caseous necrotic tissue within the center of the granuloma. *GranSim* is parameterized based on data derived from various sources including non-human primates, rabbits, mice, *in vitro*, and human studies. In this study, we excluded consideration of drug-resistant TB and only consider drug-susceptible TB, as it is the most prevalent type of TB with ~96% of the new TB cases [3]. Our model is easily adapted to include drug resistance as we have done previously [4].

## *Pharmacokinetic (PK) modeling of antibiotics*

We have previously established a PK model that is simulated within *GranSim* capturing spatial antibiotic distribution following oral doses within different tissue types, i.e., plasma, granuloma, uninvolved lung, and caseum [5, 6]. We capture plasma PK as follows: we used a compartmental model with a system of ordinary differential equations to simulate antibiotic concentrations in plasma following oral doses. Then, we simulated the permeation of antibiotics into the lungs by calculating the flux of antibiotics onto the simulation grid through lung vascular sources. Once drug is present within simulated lung tissue, antibiotics diffused through the lung tissue, bound to macromolecules (e.g., caseum, epithelial tissue etc.), and partitioned into macrophages (see <http://malthus.micro.med.umich.edu/GranSim/> for the full list of equations and code files). We calibrated PK parameters that govern these processes (e.g., diffusivity, caseum unbound fraction, cell uptake etc.) based on *in vivo* studies, such as HPLC coupled to tandem mass spectrometry (LC-MS/MS) and MALDI mass spectrometry imaging (MALDI-MSI) analyses from humans or animal models exposed to human-equivalent doses, such as NHPs or rabbits. From our previous studies, we have incorporated PK of the antibiotics isoniazid (H), rifampicin (R), pyrazinamide (Z), ethambutol (E), and moxifloxacin (M) [6-9] and in this work, we also included PK of bedaquiline (B), pretomanid (Pa), and linezolid (L) into *GranSim* as we have done previously [10].

The PK data for L is given in [10] (Fig S1). Drugs B and Pa are a part of a larger study that was performed outside the scope of this paper and those data will be published therein (unpublished data from Dr Veronique Dartois).


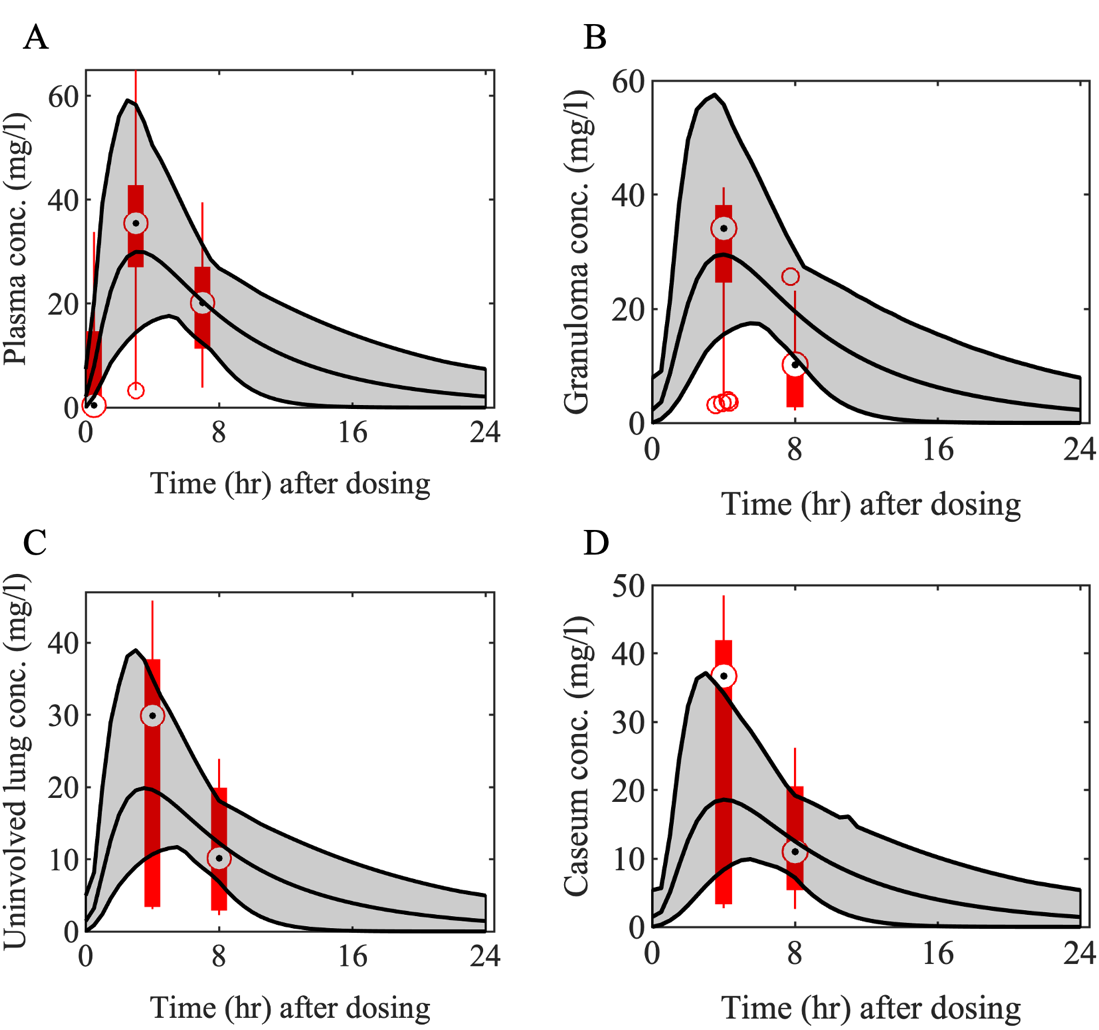


Fig. S1: **Calibration of pharmacokinetic (PK) parameters for linezolid within *GranSim****.* Panels show PK calibration of linezolid (L) to *in vivo* antibiotic concentration data using the *GranSim*-PKPD framework. Data shown is derived from different tissue types from rabbits: (A) blood plasma, (B) granuloma, (C) uninvolved lung and (D) caseum. In each panel, red box plots represent data from rabbits. The dots in the middle of the box plots represent the median, whereas the top and the bottom of the thick red column of the box plot represent 75^th^ and 25^th^ percentile, respectively. The black lines are the maximum, the mean and the minimum antibiotic concentrations from 200 *GranSim* simulations, and the grey shaded area is the area between the maximum and the minimum concentration.

Table S1: **Plasma pharmacokinetics parameter ranges for all drugs (see Appendix B in [5] for the plasma PK ODE equations)**

| Parameter name | Description | Drugs | | | | | | | |
| --- | --- | --- | --- | --- | --- | --- | --- | --- | --- |
|  |  | H | R | Z | E | M | B | Pa | L |
| k_a_ (h^-1^) | Absorption rate constant | [0.5,6.0] | [0.2,0.4] | [0.55,0.75] | [1.0,2.75] | [0.35,1.0] | [0.03,0.1] | [0.35,1.2] | [0.48,1.3] |
| Q (L/(kg.h)) | Intercompartmental clearance rate constant | [0.2,0.7] | [1.0,7.5] | [0.1,0.7] | [0.35,7.39] | [0.6,12] | [0.2,1.0] | [3,4] | [0.38,6.0] |
| V_P_ (L/kg) | Plasma volume distribution | [0.5,3.0] | [0.08,0.6] | [0.25,0.75] | [2.7,4.5] | [1.65,4.5] | [0.05,5] | [1.0,4.0] | [0.16,1.71] |
| V_Pe_ (L/kg) | Peripheral volume distribution | [25,40] | [0.05,0.2] | [0.01,0.05] | [0.08,1.0] | [0.05,0.6] | [0.1,50] | [3.5,6.0] | [0.2,1.9] |
| CL (L/(kg.h)) | Plasma clearance rate constant | [0.008,0.07] | [0.08,0.15] | [0.01,0.05] | [1.65,1.8] | [0.08,0.15] | [2,5] | [0.2,0.8] | [0.21,0.41] |

## *Pharmacodynamic (PD) modeling with PD drug interactions*

We have a well-established PD model for antibiotics including PD drug interactions within *GranSim* [5, 8]. Briefly, we use a Hill function that determines the killing rate constant (k) based on the antibiotic concentration (C) within each microgrid: $k\left( C \right)=E_{max}\frac{C^{h}}{C^{h}+{C50}^{h}}$ , where *E_max_, C50* and *h* are the maximum killing rate, the concentration to reach the half maximum killing rate (*E_max_/2*), and the Hill constant, respectively. We calibrate the constants of the Hill function (*E_max_, C50* and *h*) to *in vitro* bactericidal assays. To calibrate these constants for intracellular, extracellular and nonreplicating Mtb, we used macrophage assays, Mtb assays and assays in caseum mimic, respectively. Since the microenvironments of each type of Mtb in *GranSim* mimics the conditions in each type of assay, we did not correct the drug concentrations for binding to the macromolecules.

In this study, we calibrated PD parameters of B [11, 12], Pa [13-15] and L [12, 15, 16] for intracellular, extracellular, and nonreplicating Mtb (Fig S2 and Table S2).

Fig S2. **Calibration of pharmacodynamic (PD) parameters within *GranSim.*** Shown are the PD parameter calibrations using *GranSim* for the antibiotics (A-C) B, (D-F) Pa and (G-I) L for intracellular (A, D, and G), extracellular (B, E, and H) and nonreplicating Mtb (C, F, and I). Red circles represent *in vitro* data [11-16], whereas black lines represent model PD calibrations.

Table S2: **Pharmacodynamics model parameters of intracellular, extracellular, and nonreplicating Mtb subpopulations for bedaquiline (B), pretomanid (Pa), and linezolid (L).**

| **Bacterial subpopulation** | **Parameter** | **Bedaquiline** | **Pretomanid** | **Linezolid** |
| --- | --- | --- | --- | --- |
| **Intracellular** | Emax (1/timestep) | 0.049 | 0.0032 | 0.003 |
|  | C50 (mg/L) | 0.17 | 0.0677 | 0.3 |
|  | h | 7.4 | 2.62 | 2.6 |
| **Extracellular** | Emax (1/timestep) | 0.63 | 0.0065 | 0.0053 |
|  | C50 (mg/L) | 3.17 | 0.21 | 0.51 |
|  | h | 1.7 | 0.97 | 2.08 |
| **Nonreplicating** | Emax (1/timestep) | 0.0039 | 0.0039 | 0.0024 |
|  | C50 (mg/L) | 5.14 | 5.06 | 1.35 |
|  | h | 0.74 | 1.33 | 0.94 |

*Drug interaction modeling for combination therapy*

As we have done previously [8, 9], when more than one antibiotic is present, to capture the effects of drug interactions, we used the Hill function parameter set of the antibiotic with the highest maximal killing rate (drug 1). Then, we adjusted the concentrations of remaining antibiotics to the drug 1 parameter set, i.e., we find the concentration for each drug that resulted in the same antibiotic killing rate constant as their own parameter sets. We then calculated an effective concentration by considering the fractional inhibitory concentration of the combination (FIC) to simulate synergistic and antagonistic effects of the combinations [8, 9] (see <http://malthus.micro.med.umich.edu/GranSim/> for the full list of equations and code files). We used FIC values previously predicted by INDIGO-MTB (inferring drug interactions using chemogenomics and orthology optimized for Mtb). INDIGO-MTB is a tool that takes known drug interactions and drug transcriptomics data as inputs and predicts unknown drug interactions, i.e., FICs using a machine learning algorithm [17, 18].

Table S3: **FIC values for all combinations predicted by INDIGO-MTB and used in *GranSim***

|  | **Regimen** | **FIC** |  | **Regimen** | **FIC** |
| --- | --- | --- | --- | --- | --- |
| **2-way** | HR | 1.37493878 | **4-way** | HRZE | 0.81887465 |
|  | HZ | 1.11009417 |  | HRZB | 0.75671329 |
|  | HE | 1.1343591 |  | HRZPa | 0.62223372 |
|  | HB | 1.14967728 |  | HRZM | 0.96805236 |
|  | HPa | 1.05614823 |  | HRZL | 0.81415524 |
|  | HM | 2.01157218 |  | HREB | 0.85055197 |
|  | HL | 1.0346804 |  | HREPa | 0.69748379 |
|  | RZ | 0.9058053 |  | HREM | 1.06359541 |
|  | RE | 0.86462618 |  | HREL | 0.79340117 |
|  | RB | 1.81399312 |  | HRBPa | 0.53712875 |
|  | RPa | 0.41373443 |  | HRBM | 1.04197505 |
|  | RM | 2.27300777 |  | HRBL | 0.75070028 |
|  | RL | 0.71387791 |  | HRPaM | 0.84987228 |
|  | ZE | 0.98525798 |  | HRPaL | 0.63992778 |
|  | ZB | 0.9348166 |  | HRML | 0.94953416 |
|  | ZPa | 0.97547576 |  | HZEB | 0.84581803 |
|  | ZM | 1.9282003 |  | HZEPa | 0.79287347 |
|  | ZL | 0.88252342 |  | HZEM | 1.08162105 |
|  | EB | 0.9409951 |  | HZEL | 0.80822608 |
|  | EPa | 1.06195731 |  | HZBPa | 0.70309736 |
|  | EM | 2.05601357 |  | HZBM | 0.97915609 |
|  | EL | 0.92132403 |  | HZBL | 0.74047527 |
|  | BPa | 0.91869621 |  | HZPaM | 0.9063158 |
|  | BM | 2.30568568 |  | HZPaL | 0.77512211 |
|  | BL | 0.86227654 |  | HZML | 0.97347828 |
|  | PaM | 2.04041685 |  | HEBPa | 0.73654542 |
|  | PaL | 0.84168687 |  | HEBM | 1.03488372 |
|  | ML | 2.02801036 |  | HEBL | 0.85521411 |
| **3-way** | HRZ | 0.81227372 |  | HEPaM | 0.96281022 |
|  | HRE | 0.86685121 |  | HEPaL | 0.79769131 |
|  | HRB | 1.16305605 |  | HEML | 1.00808557 |
|  | HRPa | 0.59800284 |  | HBPaM | 0.86270763 |
|  | HRM | 1.06095552 |  | HBPaL | 0.7062173 |
|  | HRL | 0.78624612 |  | HBML | 0.9360722 |
|  | HZE | 0.89566548 |  | HPaML | 0.92113401 |
|  | HZB | 0.8244033 |  | RZEB | 0.77333118 |
|  | HZPa | 0.73215379 |  | RZEPa | 0.75599311 |
|  | HZM | 1.14987676 |  | RZEM | 1.07399294 |
|  | HZL | 0.80001987 |  | RZEL | 0.75670504 |
|  | HEB | 0.85660055 |  | RZBPa | 0.64189935 |
|  | HEPa | 0.88285925 |  | RZBM | 1.02552766 |
|  | HEM | 1.10345099 |  | RZBL | 0.68385794 |
|  | HEL | 0.84671359 |  | RZPaM | 0.9234684 |
|  | HBPa | 0.64776011 |  | RZPaL | 0.71430721 |
|  | HBM | 1.04233737 |  | RZML | 0.93226459 |
|  | HBL | 0.83725574 |  | REBPa | 0.67477613 |
|  | HPaM | 1.02009743 |  | REBM | 0.99892776 |
|  | HPaL | 0.82093419 |  | REBL | 0.69678891 |
|  | HML | 1.09735975 |  | REPaM | 0.9363944 |
|  | RZE | 0.84840369 |  | REPaL | 0.6519329 |
|  | RZB | 0.78884617 |  | REML | 0.9870801 |
|  | RZPa | 0.66558422 |  | RBPaM | 0.88671776 |
|  | RZM | 1.05843152 |  | RBPaL | 0.57508524 |
|  | RZL | 0.73831966 |  | RBML | 0.901738 |
|  | REB | 0.83904859 |  | RPaML | 0.87519058 |
|  | REPa | 0.70484057 |  | ZEBPa | 0.91765286 |
|  | REM | 1.07996797 |  | ZEBM | 1.05194238 |
|  | REL | 0.73357689 |  | ZEBL | 0.87536686 |
|  | RBPa | 0.57770934 |  | ZEPaM | 1.02632171 |
|  | RBM | 1.12304005 |  | ZEPaL | 0.84671023 |
|  | RBL | 0.73441925 |  | ZEML | 1.01302283 |
|  | RPaM | 0.88887818 |  | ZBPaM | 0.96859695 |
|  | RPaL | 0.62786615 |  | ZBPaL | 0.85166252 |
|  | RML | 0.99742627 |  | ZBML | 0.96284313 |
|  | ZEB | 0.94652946 |  | ZPaML | 0.94701814 |
|  | ZEPa | 0.90146214 |  | EBPaM | 1.04471484 |
|  | ZEM | 1.04534613 |  | EBPaL | 0.89559492 |
|  | ZEL | 0.89779738 |  | EBML | 1.02960538 |
|  | ZBPa | 0.88252066 |  | EPaML | 0.9668899 |
|  | ZBM | 1.06698779 |  | BPaML | 0.99367868 |
|  | ZBL | 0.84336153 |  |  |  |
|  | ZPaM | 0.99487638 |  |  |  |
|  | ZPaL | 0.87973725 |  |  |  |
|  | ZML | 0.89193701 |  |  |  |
|  | EBPa | 0.92955008 |  |  |  |
|  | EBM | 1.0303233 |  |  |  |
|  | EBL | 0.9182396 |  |  |  |
|  | EPaM | 1.03421695 |  |  |  |
|  | EPaL | 0.86258437 |  |  |  |
|  | EML | 0.99619078 |  |  |  |
|  | BPaM | 1.05508012 |  |  |  |
|  | BPaL | 0.89222174 |  |  |  |
|  | BML | 1.02749732 |  |  |  |
|  | PaML | 0.94885298 |  |  |  |

## *In silico* granuloma library

To test the efficacies of regimens at a granuloma scale, we first generated 5 sets of *in silico* granulomas with 200 granulomas in each set, totaling N=1000. To generate each granuloma set, we sampled 250 parameter sets using the Latin hypercube sampling (LHS) method [19] within physiologically relevant ranges previously calibrated to temporal CFU counts from 646 cynomolgous macaque granulomas [9, 20, 21]. The LHS method assumed the parameters are independent and divided parameter space into 250 equally probable intervals and samples without replacement from these intervals [19]. LHS samples the parameter space more uniformly than a random sampling method [22]. We simulated 250 parameter sets with 3 replications to address aleatory (stochastic) uncertainty [19]. We simulated each of these 750 granulomas for 300 days post-infection.

Table S4: **Host immune parameters for in silico granulomas. Timestep units represent 10-min time steps in the agent-based simulation.**

| **Parameter definition** | **Units** | **Range** |
| --- | --- | --- |
| # immune cell deaths causing compartment caseation | Number of killings for a compartment to become caseated | [12, 18] |
| Time to heal caseated compartment | Timesteps | [1386, 1647] |
| TNF threshold for causing immune cell apoptosis | Molecules | [1195, 1328] |
| Rate constant for TNF-induced apoptosis | 1/s | [1.5e-06, 7.5e-06] |
| Minimum chemokine concentration to induce chemotaxis | Molecules | [0.39, 0.51] |
| Maximum chemokine concentration to induce chemotaxis | Molecules | [397.0, 504.3] |
| Initial density of macrophages | Fraction of grid compartments | [0.005, 0.01] |
| Time between resting macrophage movements | Timesteps | [4, 6] |
| Time between active macrophage movements | Timesteps | [21, 23] |
| Time between infected macrophage movements | Timesteps | [133, 172] |
| TNF threshold to induce NFkB activation | Molecules | [74.52, 85.42] |
| Rate constant for NFkB activation | 1/s | [6.76e-06, 1.01e-05] |
| Probability resting macrophage kills extracellular Mtb |  | [0.097, 0.153] |
| Killing probability adjustment for resting macrophages with NFkB activation |  | [0.14, 0.26] |
| # bacteria to cause NFkB activation |  | [145, 256] |
| # bacteria for macrophage to become chronically infected |  | [10, 15] |
| # bacteria to cause macrophage to burst |  | [24, 41] |
| # bacteria activated macrophage can phagocytose |  | [3, 10] |
| Probability activated macrophage will will heal a caseated compartment |  | [0.004, 0.007] |
| Probability a T-cell will move to same compartment as a macrophage |  | [0.034, 0.055] |
| Probability IFNγ producing T-cell induces Fas/FasL apoptosis |  | [0.029, 0.0405] |
| Probability IFNγ producing T-cell also produces TNF |  | [0.04, 0.05] |
| Probability IFNγ producing T-cell also produces IFNγ |  | [0.29, 0.46] |
| Probability cytotoxic T-cell kills macrophage |  | [0.007, 0.010] |
| Probability cytotoxic T-cell kills a macrophage and all its intracellular bacteria |  | [0.58, 0.91] |
| Probability cytotoxic T-cell also produces TNF |  | [0.039, 0.061] |
| Probability regulatory T-cell deactivates macrophage |  | [0.0058, 0.0102] |
| Time when T-cell recruitment begins | Timesteps | [3160, 4275] |
| Time delay after T-cell recruitment begins until maximal recruitment rate | Timesteps | [770, 1196] |
| Macrophage maximal recruitment probability |  | [0.088, 0.308] |
| Macrophage threshold for recruitment by chemokines | Molecules | [0.91, 3.11] |
| Macrophage threshold for recruitment by TNF | Molecules | [0.0075, 0.0626] |
| Macrophage half sat for recruitment by TNF | Molecules | [1.84, 5.05] |
| Macrophage half sat for recruitment by chemokine | Molecules | [2.37, 4.62] |
| IFNγ producing T-cell maximal recruitment probability |  | [0.16, 0.84] |
| IFNγ producing T-cell threshold for recruitment by chemokine | Molecules | [0.037, 0.093] |
| IFNγ producing T-cell threshold for recruitment by TNF | Molecules | [0.89, 1.21] |
| IFNγ producing T-cell half sat for recruitment by TNF | Molecules | [0.75, 1.64] |
| IFNγ producing T-cell half sat for recruitment by chemokine | Molecules | [0.98, 1.52] |
| Probability a IFNγ producing T-cell is cognate |  | [0.026, 0.094] |
| Cytotoxic T-cell maximal recruitment probability |  | [0.18, 0.82] |
| Cytotoxic T-cell threshold for recruitment by chemokine | Molecules | [2.93, 4.07] |
| Cytotoxic T-cell threshold for recruitment by TNF | Molecules | [0.98, 1.52] |
| Cytotoxic T-cell half sat for recruitment by TNF | Molecules | [0.98, 1.53] |
| Cytotoxic T-cell half sat for recruitment by chemokine | Molecules | [4.76, 10.27] |
| Probability a cytotoxic T-cell is cognate |  | [0.006, 0.105] |
| Regulatory T-cell maximal recruitment probability |  | [0.18, 0.81] |
| Regulatory T-cell threshold for recruitment by chemokine | Molecules | [1.48, 2.54] |
| Regulatory T-cell threshold for recruitment by TNF | Molecules | [0.94, 2.05] |
| Regulatory T-cell half sat for recruitment by TNF | Molecules | [1.46, 2.36] |
| Regulatory T-cell half sat for recruitment by chemokine | Molecules | [0.94, 2.04] |
| Probability a regulatory T-cell is cognate |  | [0.005, 0.105] |


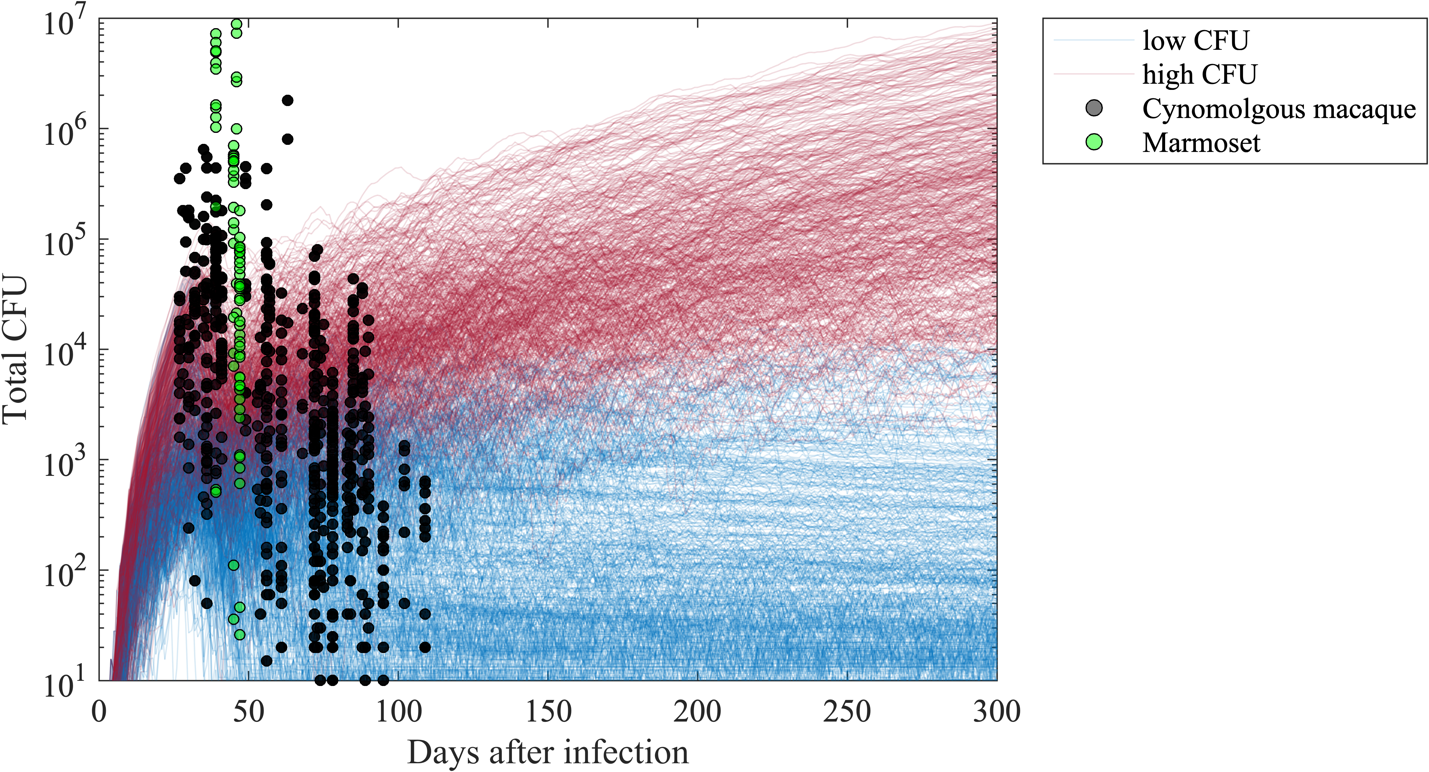


Fig S3. **CFU trends of granulomas generated in *GranSim* aligned with data derived from NHP studies.** Using *GranSim,* simulated CFU trends are shown after the start of infection. Each curve represents a simulation of one granuloma with a single parameter set. Dots are CFU counts from NHP granulomas (cynomolgous macaques and marmosets). Black dots are individual granuloma CFU counts from 42 cynomologus macaques having 646 granulomas in total [9, 20, 21], where each monkey has 2-40 granulomas (the median is 14.5, 25^th^ and 75^th^ percentiles are 9 and 20, respectively.). Green dots are individual granuloma CFU counts from 4 marmosets having 76 granulomas in total, where each monkey has 13, 7, 18 and 38 granulomas (see Methods). We categorize simulated granulomas based on their CFU trajectories into low-CFU (blue curves, N=500) and high-CFU (red curves, N=500) granulomas that represent granulomas with controlled and uncontrolled CFU burden, respectively. Note the model is flexible to, and not dependent on, this threshold cut-off.

To assess regimen efficacies between granulomas that control their bacterial load versus those that do not, we tracked the temporal CFU counts of the resulting 750 granulomas and separated them into 2 groups based on their CFU numbers and trends: low-CFU and high-CFU granulomas. Low-CFU granulomas represented granulomas mainly found in latent TB cases, i.e., granulomas that were in low, steady-state CFU levels controlled by the immune response (Fig S3). At least 1 high-CFU granuloma is likely present in active TB cases [23]. The number of CFUs in high-CFU granulomas grows uncontrollably and likely will disseminate. Briefly, we assumed low-CFU granulomas had nonzero CFUs, were less than 10^4^ at the end of simulating 300 days post-infection, and the change in increase in their CFUs was less than 50 units in the last 20 days of the simulation. We labeled granulomas as high-CFU if their CFUs fell between 10^4^ and 10^7^ units at the end of the simulation or they increased more than 50 CFUs in the last 20 days of the simulation. We picked 100 low-CFU and 100 high-CFU granulomas for each of the 5 *in silico* granuloma libraries, creating a library of 1000 granulomas (see Fig S4 for the rationale behind choosing 5 sets with 200 granulomas each). Note that our model has been calibrated to cynomolgus macaque data for 20 years. Marmoset granulomas are overlapped in Fig S3 for comparison purposes, and it is expected for simulated granulomas to not completely match marmoset granulomas, except in the high CFU case.

Marmoset Experiments

The full marmoset studies will be reported in future studies, but briefly, we describe the studies below. Breeding of *Callithrix jacchus* pairs was done in NIH facilities and all procedures were performed in accordance with the recommendations of the Guide for the Care and Use of Laboratory Animals of the National Institutes of Health. The NIAID Animal Care and Use Committee approved all experiments in Protocol LCIM-9, (Permit issued to NIH as A-4149-01). Prior to infection, male and female marmosets between 2 and 5 years of age were transferred to a BSL-3 animal facility approved for the containment of Mtb, housed in pairs, and handled as previously described [24, 25]. Marmosets were infected with Mtb H37Rv with a nose-only aerosol generated by a BANG nebulizer through a CH Technologies inhalation system (Westwood, NJ) that generated 10–25 granulomas per animal. Marmosets were treated with the drugs and combinations of drugs listed in Table 2 for 2 months beginning about 7 weeks after infection when the animals were observed to have lesions in their lung by PET/CT and weight loss had commenced as previously described [24, 26].

Data from seventy-nine marmosets treated for 2 months with one of 15 drugs/combinations listed in Table 2 (n=5 per group) or untreated (n=4) were included in this study. The drugs used were from commercial, approved for human-use, sources and administered orally by body weight as described in Table S8. Prior to the use of each drug, single and steady state dose experiments were conducted in naïve marmosets to determine a tolerable dose with exposure like that seen in humans (Table S8). Single drugs and combinations examined included: B, Pa, H, Z, R, M, BPa, PaL, BL, HZ, RZ, RM, BPaL, HRZE, and RMZE. Drug monitoring through collection of monthly blood samples (as described in [24]) during treatment did not reveal altered exposure of the single drugs when given in combination. At necropsy, samples of all identified lung lesions and 3 random pieces of apparently normal lung were collected, classified by gross pathology, segmented for histopathology and bacterial burden assessment. The portion for bacterial burden was weighed, homogenized, and plated in triplicate onto M7H11 agar supplemented with albumin, oleic acid, dextrose, and activated charcoal powder (0.4% w/v) to enumerate bacterial CFU/lesion. For each lesion collected at necropsy, the gross pathology and histology observations were used to classify the lesions into types such as cellular/fibrotic, or necrotic using previously reported histopathology methods [24, 26]. In this work, two subsets: caseous/necrotic lesions (Table S10) and fibrotic lesions/normal (Table S11) were used to evaluate drug-specific patterns.

**Analysis determining effect of the number of granuloma sets on efficacy measures**

We simulated BPaM treatment for 180 days on a varying number of granulomas to assess the number of granulomas required for the robustness of the measures we used, i.e., sterilization time and area under the sterilization curve (AUSC) (see *Measurements to assess regimen efficacies and rank* in Methods). The distribution of sterilization times (Fig S4A) did not change significantly with increasing numbers of granulomas. To allow for variability in AUSC measures, we divided granulomas into 5 groups, i.e., the case with 200 granulomas consists of 5 groups of 40 granulomas each and the case with 1000 granulomas consists of 5 groups of 200 granulomas each. The median AUSC value increased with increasing numbers of granulomas and converged at ~166 days when at least 800 granulomas are used in the analysis (Fig S4B). To ensure the robustness of our measurement, we shuffled the groups of the case with 1000 granulomas 5 times and provided evidence that the AUSC distributions do not change significantly from the 800 granuloma case (Fig S4C). Therefore, we simulated regimens on 1000 granulomas for our analysis.


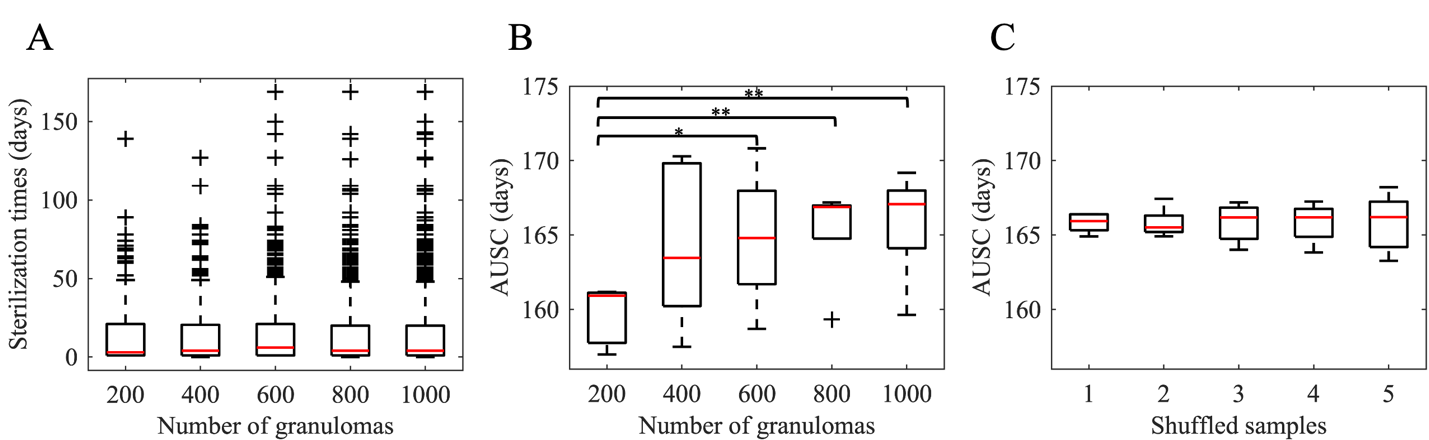


Fig S4: **The effect of numbers of granuloma sets on the accuracy of efficacy measures.** (A) The distribution of sterilization times of granuloma sets containing 200, 400, 600, 800, and 1000 granulomas after the treatment with BPaM. (B) Distribution of area under the sterilization curves (AUSC) for varying numbers of granulomas divided by 5 groups. AUSCs are calculated separately for each group. (C) Five groups with 200 granulomas each, 1000 in total, are shuffled 5 times and AUSCs are calculated for each group. The central red lines in box plots represent the median, whereas the bottom and the top edges of boxes represent 25th and 75th percentiles, respectively.


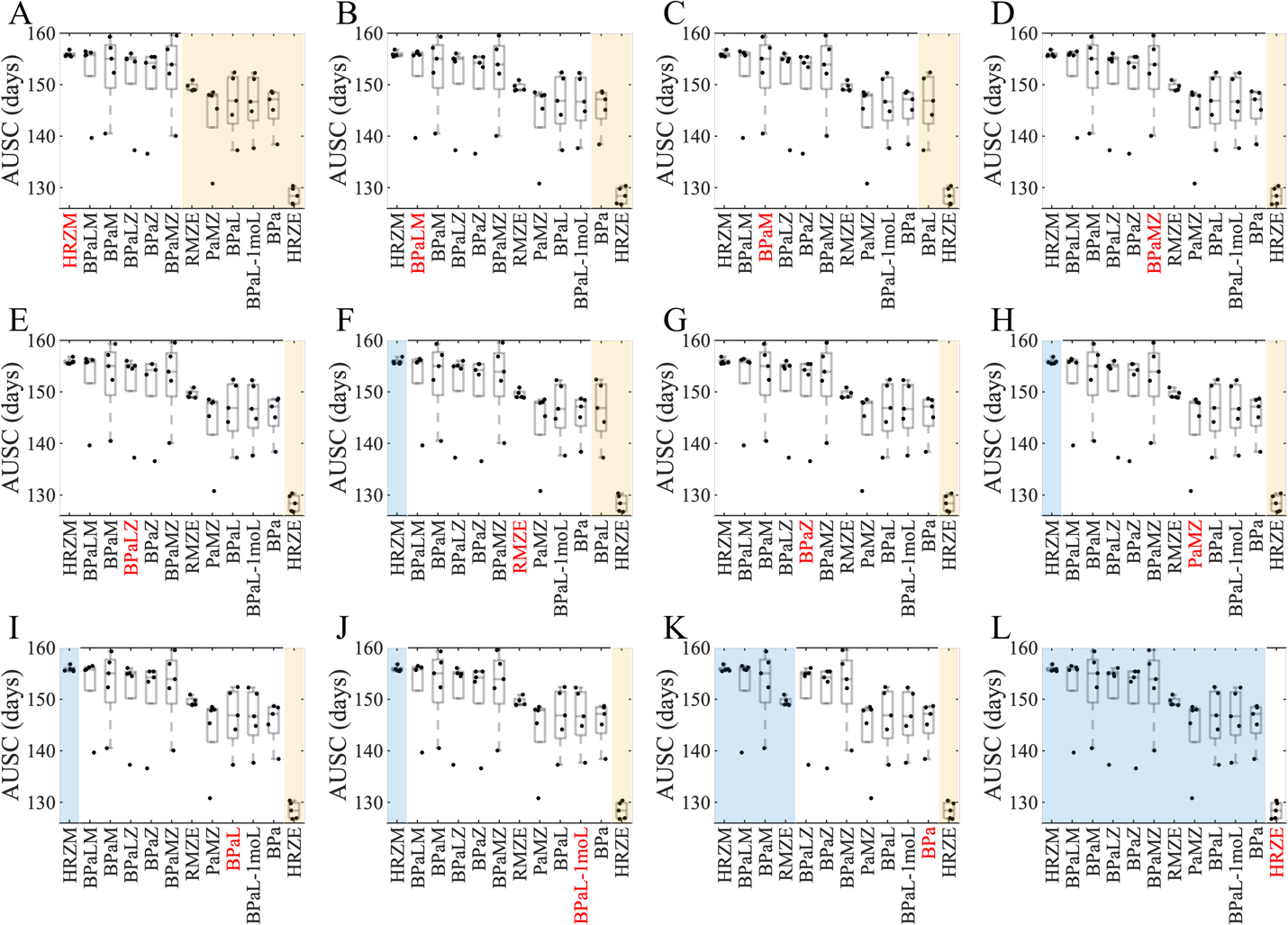


Fig S5: **AUSC plots of regimens compared with the reference regimen for high-CFU granulomas only**. We derived boxplots for the areas under the sterilization curves (AUSCs) upon treatment with each regimen, considering high-CFU granulomas only. Each dot represents the AUSC of one group of 100 granulomas. In each panel, the regimen in red (reference regimen) is compared pairwise to the regimens in black. The regimens in the yellow-shaded area indicate that these regimens have significantly lower AUSCs than the reference regimen. Likewise, the regimens in the blue-shaded area mean that these regimens have significantly higher AUSCs than the reference regimen. The difference between the AUSCs of the regimens with a white background and the reference regimen is insignificant (one-tailed, pairwise t-test, p<0.05).


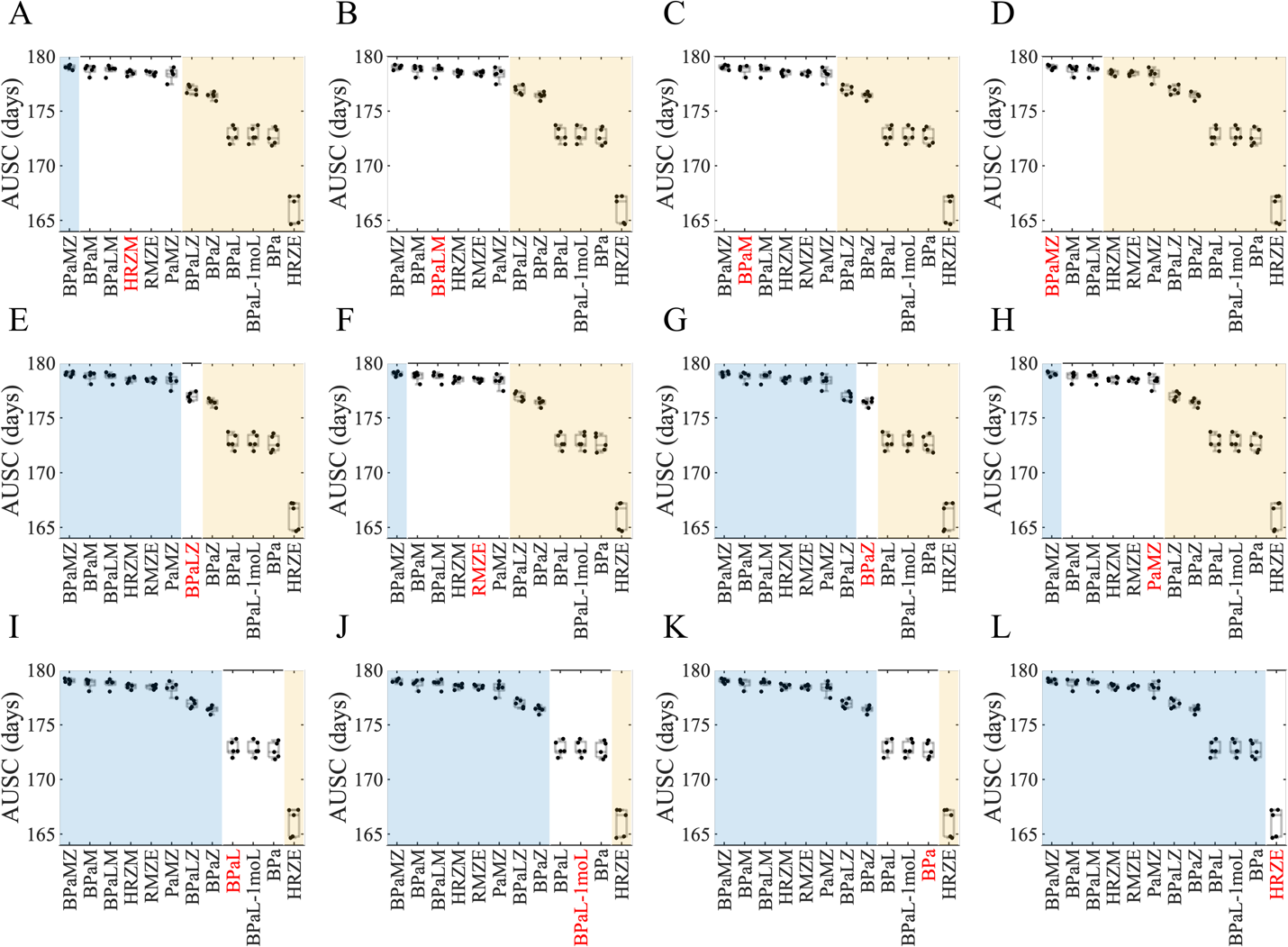


Fig S6: **AUSC plots of regimens compared to a reference regimen for low-CFU granulomas only**. We derived boxplots for the areas under the sterilization curves (AUSCs) upon treatment with each regimen, considering low-CFU granulomas only. Each dot represents the AUSC of one group of 100 granulomas. In each panel, the regimen in red (reference regimen) is compared pairwise to the regimens in black. The regimens in the yellow-shaded area indicate that these regimens have significantly lower AUSCs than the reference regimen. Likewise, the regimens in the blue-shaded area mean that these regimens have significantly higher AUSCs than the reference regimen. The difference between the AUSCs of the regimens with a white background and the reference regimen is insignificant (one-tailed, pairwise t-test, p<0.05).

Table S5: **Rankings based on scores for each regimen set on the total 1000 simulated granuloma set**. Shown are both ranking scores and rankings of each regimen in the regimen set, considering high- and low-CFU granulomas. Dark purple-shaded regimens are candidates for the most potent regimens and regimens with the white background are candidates for the least potent regimens. The regimens in the light purple-shaded area are those regimens for which AUSCs are higher (and lower) than at least one regimen in the regimen set.

| **Reference regimen** | **Rank** | **Nr of regimens significantly worse than the reference regimen (w)** | **Regimens that are significantly worse than the reference regimen** | **Nr of regimens significantly better than the reference regimen (b)** | **Regimens that are significantly better than the reference regimen** | **Score (w-b)** | **Reference regimen is not significantly different than** |
| --- | --- | --- | --- | --- | --- | --- | --- |
| **HRZM** | 1 | 7 | HRZE, RMZE, BPaL, BPa, BPaZ, PaMZ, BPaL-1moL | 0 | - | 7 | BPaM, BPaLZ, BPaMZ, BPaLM |
| **BPaM** | 2 | 5 | HRZE, BPaL, BPa, PaMZ, BPaL-1moL | 0 | - | 5 | RMZE, HRZM, BPaZ, BPaLZ, BPaMZ, BPaLM |
| **BPaMZ** | 2 | 5 | HRZE, BPaL, BPa, PaMZ, BPaL-1moL | 0 | - | 5 | RMZE, HRZM, BPaM, BPaZ, BPaLZ, BPaLM |
| **BPaLM** | 2 | 5 | HRZE, BPaL, BPa, PaMZ, BPaL-1moL | 0 | - | 5 | RMZE, HRZM, BPaM, BPaZ, BPaLZ, BPaMZ |
| **BPaLZ** | 5 | 4 | HRZE, BPaL, BPa, BPaL-1moL | 0 | - | 4 | RMZE, HRZM, BPaM, BPaZ, PaMZ, BPaMZ, BPaLM |
| **RMZE** | 6 | 4 | HRZE, BPaL, BPa, BPaL-1moL | 1 | HRZM | 3 | BPaM, BPaZ, BPaLZ, PaMZ, BPaMZ, BPaLM |
| **BPaZ** | 7 | 2 | HRZE, BPa | 1 | HRZM | 1 | RMZE, BPaL, BPaM, BPaLZ, PaMZ, BPaMZ, BPaL-1moL, BPaLM |
| **PaMZ** | 8 | 1 | HRZE | 4 | HRZM, BPaM, BPaMZ, BPaLM | -3 | RMZE, BPaL, BPa, BPaZ, BPaLZ, BPaL-1moL |
| **BPaL** | 9 | 1 | HRZE | 6 | RMZE, HRZM, BPaM, BPaLZ, BPaMZ, BPaLM | -5 | BPa, BPaZ, PaMZ, BPaL-1moL |
| **BPaL-1moL** | 9 | 1 | HRZE | 6 | RMZE, HRZM, BPaM, BPaLZ, BPaMZ, BPaLM | -5 | BPaL, BPa, BPaZ, PaMZ |
| **BPa** | 11 | 1 | HRZE | 7 | RMZE, HRZM, BPaM, BPaZ, BPaLZ, BPaMZ, BPaLM | -6 | BPaL, PaMZ, BPaL-1moL |
| **HRZE** | 12 | 0 | - | 11 | RMZE, BPaL, BPa, HRZM, BPaM, BPaZ, BPaLZ, PaMZ, BPaMZ, BPaL-1moL, BPaLM | -11 | - |

Table S6: **Rankings based on scores for each regimen set on the total 1000 simulated granuloma set**. Shown are both ranking scores and rankings of each regimen in the regimen set, considering high-CFU granulomas only. Dark purple-shaded regimens are candidates for the most potent regimens and regimens with the white background are candidates for the least potent regimens. The regimens in the light purple-shaded area are those regimens for which AUSCs are higher (and lower) than at least one regimen in the regimen set.

| **Reference regimen** | **Rank** | **Nr of regimens significantly worse than the reference regimen (w)** | **Regimens that are significantly worse than the reference regimen** | **Nr of regimens significantly better than the reference regimen (b)** | **Regimens that are significantly better than the reference regimen** | **Score (w-b)** | **Reference regimen is not significantly different than** |
| --- | --- | --- | --- | --- | --- | --- | --- |
| **HRZM** | 1 | 6 | HRZE, RMZE, BPaL, BPa, PaMZ, BPaL-1moL | 0 | - | 6 | BPaM, BPaZ, BPaLZ, BPaMZ, BPaLM |
| **BPaM** | 2 | 2 | HRZE, BPa | 0 | - | 2 | RMZE, BPaL, HRZM, BPaZ, BPaLZ, PaMZ, BPaMZ, BPaL-1moL, BPaLM |
| **BPaLM** | 2 | 2 | HRZE, BPa | 0 | - | 2 | RMZE, BPaL, HRZM, BPaM, BPaZ, BPaLZ, PaMZ, BPaMZ, BPaL-1moL |
| **BPaZ** | 4 | 1 | HRZE | 0 | - | 1 | RMZE, BPaL, BPa, HRZM, BPaM, BPaLZ, PaMZ, BPaMZ, BPaL-1moL, BPaLM |
| **BPaLZ** | 4 | 1 | HRZE | 0 | - | 1 | RMZE, BPaL, BPa, HRZM, BPaM, BPaZ, PaMZ, BPaMZ, BPaL-1moL, BPaLM |
| **BPaMZ** | 4 | 1 | HRZE | 0 | - | 1 | RMZE, BPaL, BPa, HRZM, BPaM, BPaZ, BPaLZ, PaMZ, BPaL-1moL, BPaLM |
| **RMZE** | 4 | 2 | HRZE, BPa | 1 | HRZM | 1 | BPaL, BPaM, BPaZ, BPaLZ, PaMZ, BPaMZ, BPaL-1moL, BPaLM |
| **BPaL** | 8 | 1 | HRZE | 1 | HRZM | 0 | RMZE, BPa, BPaM, BPaZ, BPaLZ, PaMZ, BPaMZ, BPaL-1moL, BPaLM |
| **PaMZ** | 8 | 1 | HRZE | 1 | HRZM | 0 | RMZE, BPaL, BPa, BPaM, BPaZ, BPaLZ, BPaMZ, BPaL-1moL, BPaLM |
| **BPaL-1moL** | 8 | 1 | HRZE | 1 | HRZM | 0 | RMZE, BPaL, BPa, BPaM, BPaZ, BPaLZ, PaMZ, BPaMZ, BPaLM |
| **BPa** | 11 | 1 | HRZE | 4 | RMZE, HRZM, BPaM, BPaLM | -3 | BPaL, BPaZ, BPaLZ, PaMZ, BPaMZ, BPaL-1moL |
| **HRZE** | 12 | 0 | - | 11 | RMZE, BPaL, BPa, HRZM, BPaM, BPaZ, BPaLZ, PaMZ, BPaMZ, BPaL-1moL, BPaLM | -11 | - |

Table S7: **Rankings based on scores for each regimen set on the total 1000 simulated granuloma set**. Shown are both ranking scores and rankings of each regimen in the regimen set, considering low-CFU granulomas only. Dark purple-shaded regimens are candidates for the most potent regimens and regimens with the white background are candidates for the least potent regimens. The regimens in the light purple-shaded area are those regimens for which AUSCs are higher (and lower) than at least one regimen in the regimen set.

| **Reference regimen** | **Rank** | **Nr of regimens significantly worse than the ref regimen (w)** | **Regimens that are significantly worse than the reference regimen** | **Nr of regimens significantly better than the ref regimen (b)** | **Regimens that are significantly better than the reference regimen** | **Score (w-b)** | **Reference regimen is not significantly different than** |
| --- | --- | --- | --- | --- | --- | --- | --- |
| **BPaMZ** | 1 | 9 | HRZE, RMZE, BPaL, BPa, HRZM, BPaZ, BPaLZ, PaMZ, BPaL-1moL | 0 | - | 9 | BPaM, BPaLM |
| **BPaM** | 2 | 6 | HRZE, BPaL, BPa, BPaZ, BPaLZ, BPaL-1moL | 0 | - | 6 | RMZE, HRZM, PaMZ, BPaMZ, BPaLM |
| **BPaLM** | 2 | 6 | HRZE, BPaL, BPa, BPaZ, BPaLZ, BPaL-1moL | 0 | - | 6 | RMZE, HRZM, BPaM, PaMZ, BPaMZ |
| **RMZE** | 4 | 6 | HRZE, BPaL, BPa, BPaZ, BPaLZ, BPaL-1moL | 1 | BPaMZ | 5 | HRZM, BPaM, PaMZ, BPaLM |
| **HRZM** | 4 | 6 | HRZE, BPaL, BPa, BPaZ, BPaLZ, BPaL-1moL | 1 | BPaMZ | 5 | RMZE, BPaM, PaMZ, BPaLM |
| **PaMZ** | 4 | 6 | HRZE, BPaL, BPa, BPaZ, BPaLZ, BPaL-1moL | 1 | BPaMZ | 5 | RMZE, HRZM, BPaM, BPaLM |
| **BPaLZ** | 7 | 5 | HRZE, BPaL, BPa, BPaZ, BPaL-1moL | 6 | RMZE, HRZM, BPaM, PaMZ, BPaMZ, BPaLM | -1 | - |
| **BPaZ** | 8 | 4 | HRZE, BPaL, BPa, BPaL-1moL | 7 | RMZE, HRZM, BPaM, BPaLZ, PaMZ, BPaMZ, BPaLM | -3 | - |
| **BPaL** | 9 | 1 | HRZE | 8 | RMZE, HRZM, BPaM, BPaZ, BPaLZ, PaMZ, BPaMZ, BPaLM | -7 | BPa, BPaL-1moL |
| **BPa** | 9 | 1 | HRZE | 8 | RMZE, HRZM, BPaM, BPaZ, BPaLZ, PaMZ, BPaMZ, BPaLM | -7 | BPaL, BPaL-1moL |
| **BPaL-1moL** | 9 | 1 | HRZE | 8 | RMZE, HRZM, BPaM, BPaZ, BPaLZ, PaMZ, BPaMZ, BPaLM | -7 | BPaL, BPa |
| **HRZE** | 12 | 0 | - | 11 | RMZE, BPaL, BPa, HRZM, BPaM, BPaZ, BPaLZ, PaMZ, BPaMZ, BPaL-1moL, BPaLM | -11 | - |

Table S8. **Drug doses and formulations used in marmosets and steady state exposure (AUC) achieved.**

| Drug^@^ | Oral Human Dose and AUC_0-24h_ (µg*h/mL) ^1^ | Marmoset Dose  (for 2 months) | Oral formulation for marmosets | Marmoset AUC_0-24h_  (ug*h/mL) range^1^ |
| --- | --- | --- | --- | --- |
| Linezolid (L) | 600-1200 mg daily  AUC_600mg_: 106.8 (36.5) ^2^  AUC_1200mg_: 287.7 (30.4) ^2^ [27] | 20 mg/kg daily | in 10% (v/v) water and 90% (v/v) OraSweet^(v/v) and 2% flavor (v/v) | 140-280 |
| Pretomanid (Pa) | 200 mg daily  AUC: 43 ± 13.1 SD [28] | 5 mg/kg daily | 20% (w/v) Captisol, 50% (v/v) OraSweet, 49% (v/v) water-saccharin (20% w/v saccharin) and 1% (v/v) flavoring | 33-41 |
| Bedaquiline (B)# | 400 mg daily for 14 d, 200 mg 3 x wk for 5 weeks  AUC: 41.5 (28.64, 52.25)^3^ [29]  C_avg_: 1.7 ± 0.7 µg/mL SD [30] | 8 mg/kg x 14 d, 6 mg/kg, 3 x wk^4^ | 100 mg tablets powdered and suspended in a syrup composed of 1:1 OraPlus/OraSweet^ (v/v) and 2% flavor (v/v) | 33-64  C_avg_: 1.7 ±  0.5 µg/mL SD |
| Moxifloxacin | 400 mg daily  AUC: 38.7 (21.9, 69.6)^5^ [31] | 45 mg/kg daily | 94% (v/v) H_2_0 + DexSacch (5% (w/v) Dextrose and 15% (w/v) Saccharin) and 4% (v/v) flavor | 16-53  (Median 34) |
| ^@^Isoniazid, Rifampicin, Pyrazinamide and Ethambutol were sourced, dosed daily, and had AUCs as previously described [24]. Single drugs and combinations examined included: B, Pa, H, Z, R, M, BPa, PaL, BL, HZ, RZ, RM, BPaL, HRZE, and RMZE. Drug monitoring during treatment did not reveal altered exposure of the single drugs when given in combination. | | | | |
| # Marmosets were given bedaquiline 30 min after a daily dose of aminobenzotriazole 20 mg/kg; dosing was after feeding with fat supplementation; Des-BDQ metabolite C_avg_: 2.5 µg/mL ± 0.49 µg/mL | | | | |
| ^1^ AUCs are measured at steady state over 0-24h timespan. | | | | |
| ^2^ Values are given as the geometric mean and percent coefficient of variation (% CV). | | | | |
| ^3^ AUC is given as the median, 25^th^ and 75^th^ percentiles. | | | | |
| ^4^ Marmoset bedaquiline dose was chosen to match the proportional concentration and activity of the major metabolite des-BDQ relative to bedaquiline in humans. Potential differences in protein binding between species were not accounted for when selecting human-equivalent doses. | | | | |
| ^5^ AUC is given as the median, 2.5^th^ and 97.5^th^ percentiles. | | | | |
| ^ OraPlus/OraSweet and flavoring (Perrigo, Grand Rapids MI) | | | | |


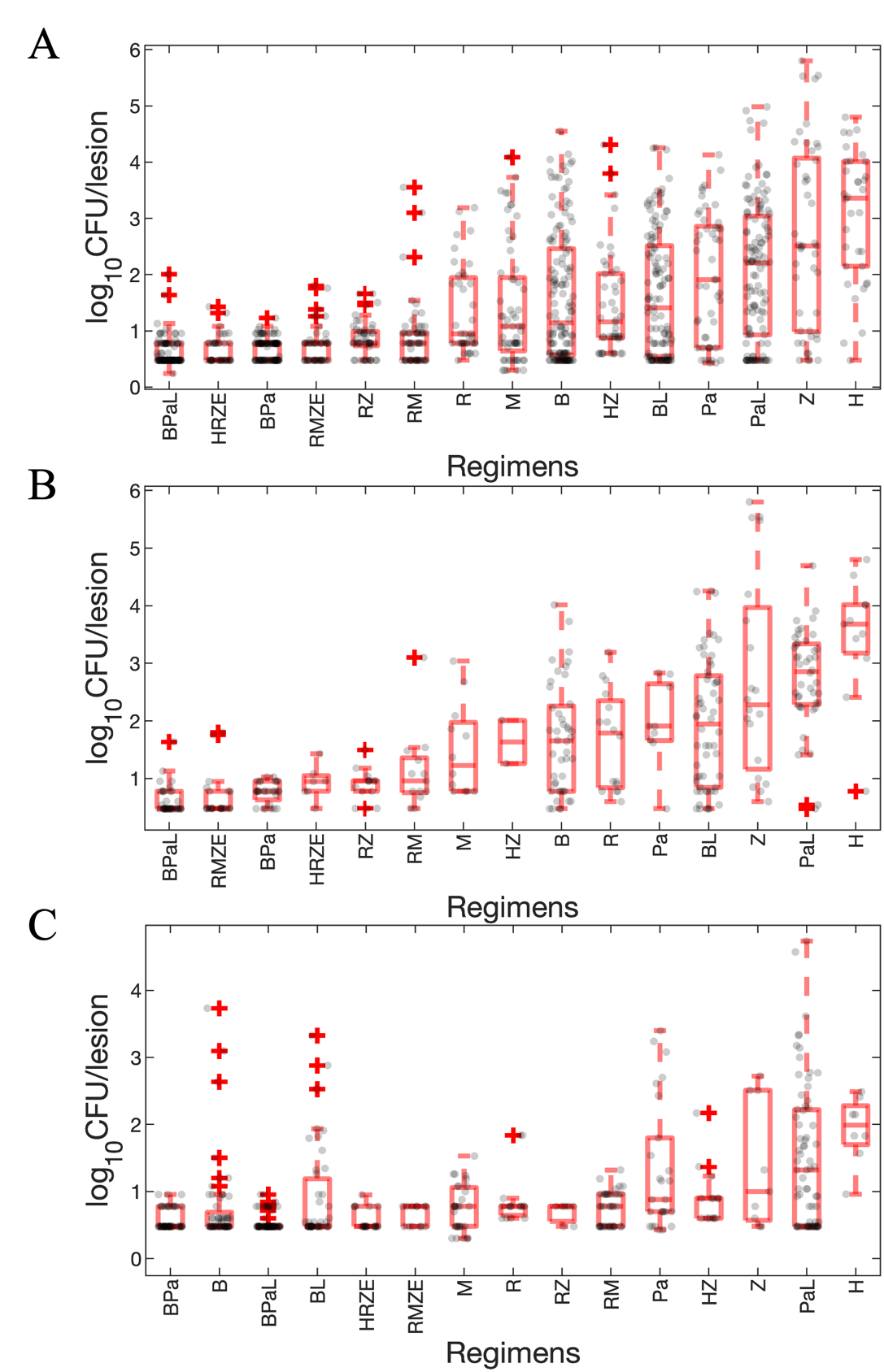


Fig S7: **Lesion-scale CFU counts from marmoset granulomas that are treated with various regimens for 60 days, considering (A) all tissue types, (B) only necrotic/caseous tissue types and (C) only fibrotic/normal tissue types.** The hosts and lesions with drug resistant Mtb are excluded from the dataset. The central line in each box represents the median, the top and bottom edges of the box represent 25^th^ and 75^th^ percentile, respectively. Boxplots are ordered based on the medians of CFU counts for each regimen.

Table S9: **Scores and ranks of each regimen from marmoset studies based on our ranking method considering only granuloma-level data from all tissue/granuloma types of marmosets.** Dark purple-shaded regimens are candidates for the most potent regimens and regimens with the white background are candidates for the least potent

regimens. The regimens in the light purple-shaded area are those regimens for which AUSCs are higher (and lower)

than at least one regimen in the regimen set.

| **Reference regimen** | **Rank** | **Nr of regimens significantly worse than the reference regimen (w)** | **Regimens that are significantly worse than the reference regimen** | **Nr of regimens significantly better than the reference regimen (b)** | **Regimens that are significantly better than the reference regimen** | **Score (w-b)** | | **Reference regimen is not significantly different than** |
| --- | --- | --- | --- | --- | --- | --- | --- | --- |
| **BPaL** | 1 | 13 | RMZE, H, M, R, Z, HZ, RZ, RM, Pa, BPa, B, BL, PaL | 0 | - | 13 | HRZE | |
| **HRZE** | 2 | 11 | H, M, R, Z, HZ, RZ, RM, Pa, B, BL, PaL | 0 | - | 11 | RMZE, BPa, BPaL | |
| **BPa** | 3 | 11 | H, M, R, Z, HZ, RZ, RM, Pa, B, BL, PaL | 1 | BPaL | 10 | RMZE, HRZE | |
| **RMZE** | 4 | 10 | H, M, R, Z, HZ, RZ, Pa, B, BL, PaL | 1 | BPaL | 9 | HRZE, RM, BPa | |
| **RM** | 5 | 9 | H, M, R, Z, HZ, Pa, B, BL, PaL | 3 | HRZE, BPa, BPaL | 6 | RMZE, RZ | |
| **RZ** | 6 | 9 | H, M, R, Z, HZ, Pa, B, BL, PaL | 4 | RMZE, HRZE, BPa, BPaL | 5 | RM | |
| **M** | 7 | 4 | H, Z, Pa, PaL | 6 | RMZE, HRZE, RZ, RM, BPa, BPaL | -2 | R, HZ, B, BL | |
| **R** | 7 | 4 | H, Z, Pa, PaL | 6 | RMZE, HRZE, RZ, RM, BPa, BPaL | -2 | M, HZ, B, BL | |
| **HZ** | 7 | 4 | H, Z, Pa, PaL | 6 | RMZE, HRZE, RZ, RM, BPa, BPaL | -2 | M, R, B, BL | |
| **B** | 7 | 4 | H, Z, Pa, PaL | 6 | RMZE, HRZE, RZ, RM, BPa, BPaL | -2 | M, R, HZ, BL | |
| **BL** | 7 | 4 | H, Z, Pa, PaL | 6 | RMZE, HRZE, RZ, RM, BPa, BPaL | -2 | M, R, HZ, B | |
| **Pa** | 12 | 2 | H, Z | 11 | RMZE, HRZE, M, R, HZ, RZ, RM, BPa, B, BPaL, BL | -9 | PaL | |
| **PaL** | 12 | 2 | H, Z | 11 | RMZE, HRZE, M, R, HZ, RZ, RM, BPa, B, BPaL, BL | -9 | Pa | |
| **H** | 14 | 0 | - | 13 | RMZE, HRZE, M, R, HZ, RZ, RM, Pa, BPa, B, BPaL, BL, PaL | -13 | Z | |
| **Z** | 14 | 0 | - | 13 | RMZE, HRZE, M, R, HZ, RZ, RM, Pa, BPa, B, BPaL, BL, PaL | -13 | H | |

Table S10: **Scores and ranks of each regimen from marmoset studies based on our ranking method, breaking down the analyses to only include granuloma-level data from necrotic/caseous tissue types of marmosets.** Dark purple-shaded regimens are candidates for the most potent regimens and regimens with the white background are candidates for the least potent regimens. The regimens in the light purple-shaded area are those regimens for which AUSCs are higher (and lower) than at least one regimen in the regimen set.

| **Reference regimen** | **Rank** | **Nr of regimens significantly worse than the reference regimen (w)** | **Regimens that are significantly worse than the reference regimen** | **Nr of regimens significantly better than the reference regimen (b)** | **Regimens that are significantly better than the reference regimen** | **Score (w-b)** | **Reference regimen is not significantly different than** |
| --- | --- | --- | --- | --- | --- | --- | --- |
| **BPaL** | 1 | 13 | HRZE, H, M, R, Z, HZ, RZ, RM, Pa, BPa, B, BL, PaL | 0 | - | 13 | RMZE |
| **RMZE** | 2 | 11 | H, M, R, Z, HZ, RZ, RM, Pa, B, BL, PaL | 0 | - | 11 | HRZE, BPa, BPaL |
| **BPa** | 2 | 12 | HRZE, H, M, R, Z, HZ, RZ, RM, Pa, B, BL, PaL | 1 | BPaL | 11 | RMZE |
| **HRZE** | 4 | 9 | H, M, R, Z, HZ, Pa, B, BL, PaL | 2 | BPa, BPaL | 7 | RMZE, RZ, RM |
| **RZ** | 5 | 9 | H, M, R, Z, HZ, Pa, B, BL, PaL | 3 | RMZE, BPa, BPaL | 6 | HRZE, RM |
| **RM** | 6 | 7 | H, R, Z, Pa, B, BL, PaL | 3 | RMZE, BPa, BPaL | 4 | HRZE, M, HZ, RZ |
| **M** | 7 | 3 | H, Z, PaL | 5 | RMZE, HRZE, RZ, BPa, BPaL | -2 | R, HZ, RM, Pa, B, BL |
| **R** | 8 | 3 | H, Z, PaL | 6 | RMZE, HRZE, RZ, RM, BPa, BPaL | -3 | M, HZ, Pa, B, BL |
| **HZ** | 8 | 2 | H, PaL | 5 | RMZE, HRZE, RZ, BPa, BPaL | -3 | M, R, Z, RM, Pa, B, BL |
| **B** | 8 | 3 | H, Z, PaL | 6 | RMZE, HRZE, RZ, RM, BPa, BPaL | -3 | M, R, HZ, Pa, BL |
| **BL** | 8 | 3 | H, Z, PaL | 6 | RMZE, HRZE, RZ, RM, BPa, BPaL | -3 | M, R, HZ, Pa, B |
| **Pa** | 12 | 2 | H, PaL | 6 | RMZE, HRZE, RZ, RM, BPa, BPaL | -4 | M, R, Z, HZ, B, BL |
| **Z** | 13 | 0 | - | 10 | RMZE, HRZE, M, R, RZ, RM, BPa, B, BPaL, BL | -10 | H, HZ, Pa, PaL |
| **PaL** | 14 | 1 | H | 12 | RMZE, HRZE, M, R, HZ, RZ, RM, Pa, BPa, B, BPaL, BL | -11 | Z |
| **H** | 15 | 0 | - | 13 | RMZE, HRZE, M, R, HZ, RZ, RM, Pa, BPa, B, BPaL, BL, PaL | -13 | Z |

Table S11: **Scores and ranks of each regimen from marmoset studies based on our ranking method, breaking down the analyses to only include granuloma-level data from fibrotic/normal tissue types of marmosets.** Dark purple-shaded regimens are candidates for the most potent regimens and regimens with the white background are candidates for the least potent regimens. The regimens in the light purple-shaded area are those regimens for which AUSCs are higher (and lower) than at least one regimen in the regimen set.

| **Reference regimen** | **Rank** | **Nr of regimens significantly worse than the ref regimen (w)** | **Regimens that are significantly worse than the reference regimen** | **Nr of regimens significantly better than the ref regimen (b)** | **Regimens that are significantly better than the reference regimen** | **Score (w-b)** | **Reference regimen is not significantly different than** |
| --- | --- | --- | --- | --- | --- | --- | --- |
| **BPaL** | 1 | 12 | RMZE, H, M, R, Z, HZ, RZ, RM, Pa, B, BL, PaL | 0 | - | 12 | HRZE, BPa |
| **BPa** | 2 | 11 | H, M, R, Z, HZ, RZ, RM, Pa, B, BL, PaL | 0 | - | 11 | RMZE, HRZE, BPaL |
| **HRZE** | 3 | 9 | H, M, R, Z, HZ, RZ, RM, Pa, PaL | 0 | - | 9 | RMZE, BPa, B, BPaL, BL |
| **RMZE** | 4 | 6 | H, R, Z, HZ, Pa, PaL | 1 | BPaL | 5 | HRZE, M, RZ, RM, BPa, B, BL |
| **B** | 5 | 4 | H, Z, Pa, PaL | 0 | - | 4 | RMZE, HRZE, M, R, HZ, RZ, RM, BPa, BPaL, BL |
| **BL** | 5 | 4 | H, Z, Pa, PaL | 0 | - | 4 | RMZE, HRZE, M, R, HZ, RZ, RM, BPa, B, BPaL |
| **RM** | 7 | 5 | H, Z, HZ, Pa, PaL | 3 | HRZE, BPa, BPaL | 2 | RMZE, M, R, RZ, B, BL |
| **M** | 8 | 4 | H, Z, Pa, PaL | 3 | HRZE, BPa, BPaL | 1 | RMZE, R, HZ, RZ, RM, B, BL |
| **RZ** | 9 | 3 | H, Z, PaL | 3 | HRZE, BPa, BPaL | 0 | RMZE, M, R, HZ, RM, Pa, B, BL |
| **R** | 10 | 3 | H, Z, PaL | 4 | RMZE, HRZE, BPa, BPaL | -1 | M, HZ, RZ, RM, Pa, B, BL |
| **HZ** | 11 | 2 | H, PaL | 5 | RMZE, HRZE, RM, BPa, BPaL | -3 | M, R, Z, RZ, Pa, B, BL |
| **Pa** | 12 | 0 | - | 8 | RMZE, HRZE, M, RM, BPa, B, BPaL, BL | -8 | H, R, Z, HZ, RZ, PaL |
| **Z** | 13 | 0 | - | 10 | RMZE, HRZE, M, R, RZ, RM, BPa, B, BPaL, BL | -10 | H, HZ, Pa, PaL |
| **H** | 14 | 0 | - | 11 | RMZE, HRZE, M, R, HZ, RZ, RM, BPa, B, BPaL, BL | -11 | Z, Pa, PaL |
| **PaL** | 14 | 0 | - | 11 | RMZE, HRZE, M, R, HZ, RZ, RM, BPa, B, BPaL, BL | -11 | H, Z, Pa |

**
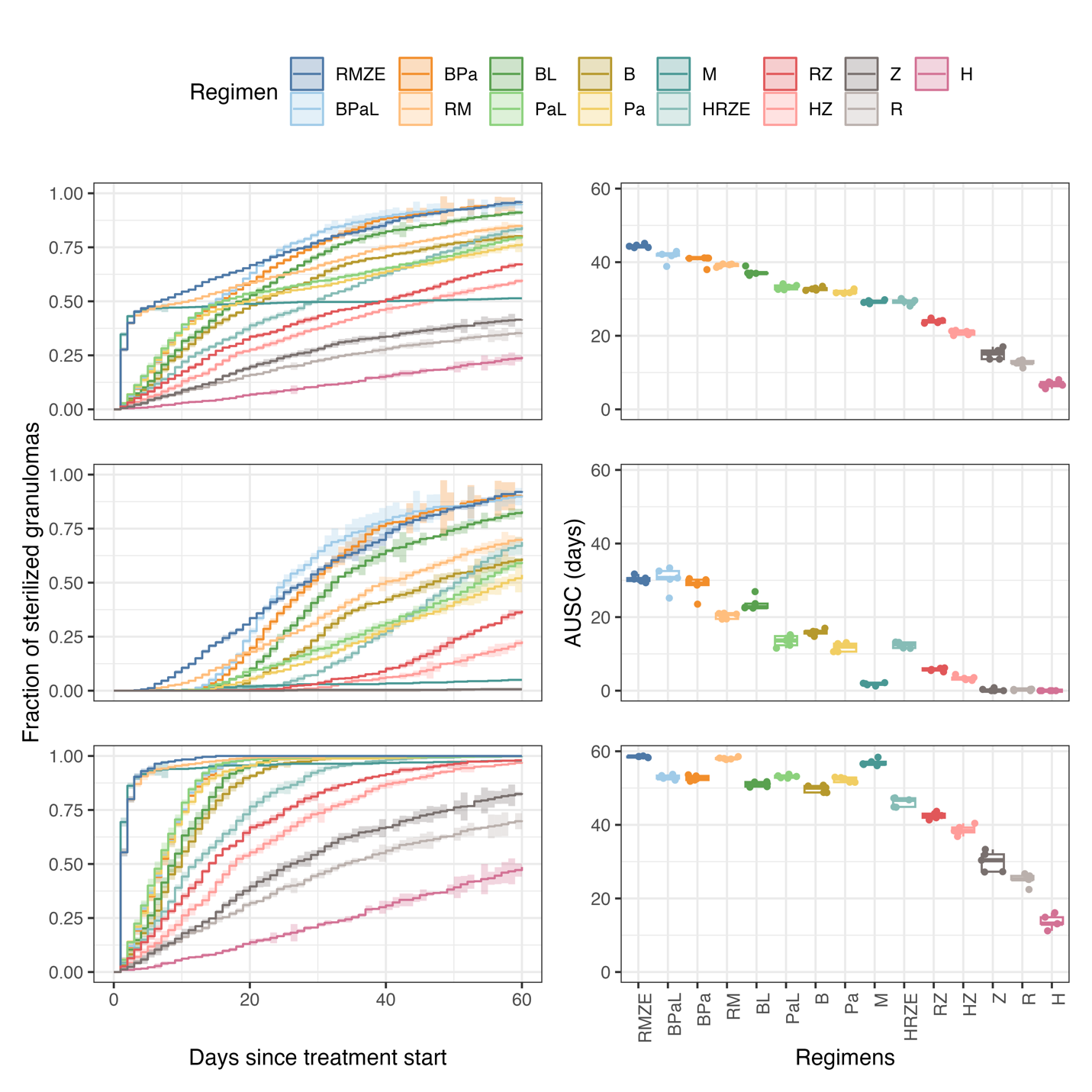
**

Fig S8: **Regimen predictions using *GranSim* with Marmoset study singles and combinations**. We show predictions when granulomas are treated with the regimens taken from the marmoset study for comparison. (A, C, E) Fraction of sterilized granulomas over the course of treatment with regimens of interest and (B, D, F) boxplots for the areas under the sterilization curves (AUSCs) considering all granulomas (A and B), high-CFU granulomas (C and D), and low-CFU granulomas (E and F).

Table S12: **Rankings based on scores for each regimen set on the total 1000 simulated granuloma set**. Shown are both ranking scores and rankings of each regimen in the regimen set, considering high- and low-CFU granulomas. Dark purple-shaded regimens are candidates for the most potent regimens and regimens with the white background are candidates for the least potent regimens. The regimens in the light purple-shaded area are those regimens for which AUSCs are higher (and lower) than at least one regimen in the regimen set.

| **Reference regimen** | **Rank** | **Nr of regimens significantly worse than the reference regimen (w)** | **Regimens that are significantly worse than the reference regimen** | **Nr of regimens significantly better than the reference regimen (b)** | **Regimens that are significantly better than the reference regimen** | **Score (w-b)** | **Reference regimen is not significantly different than** |
| --- | --- | --- | --- | --- | --- | --- | --- |
| **RMZE** | 1 | 13 | HRZE, BPaL, H, HZ, M, RZ, R, RM, BL, Z, Pa, PaL, B | 0 | - | 13 | BPa |
| **BPa** | 2 | 12 | HRZE, H, HZ, M, RZ, R, RM, BL, Z, Pa, PaL, B | 0 | - | 12 | RMZE, BPaL |
| **BPaL** | 3 | 12 | HRZE, H, HZ, M, RZ, R, RM, BL, Z, Pa, PaL, B | 1 | RMZE | 11 | BPa |
| **BL** | 4 | 11 | HRZE, H, HZ, M, RZ, R, RM, Z, Pa, PaL, B | 3 | RMZE, BPaL, BPa | 8 | - |
| **RM** | 5 | 10 | HRZE, H, HZ, M, RZ, R, Z, Pa, PaL, B | 4 | RMZE, BPaL, BPa, BL | 6 | - |
| **HRZE** | 6 | 7 | H, HZ, M, RZ, R, Z, Pa | 5 | RMZE, BPaL, BPa, RM, BL | 2 | PaL, B |
| **PaL** | 6 | 7 | H, HZ, M, RZ, R, Z, Pa | 5 | RMZE, BPaL, BPa, RM, BL | 2 | HRZE, B |
| **B** | 6 | 7 | H, HZ, M, RZ, R, Z, Pa | 5 | RMZE, BPaL, BPa, RM, BL | 2 | HRZE, PaL |
| **Pa** | 9 | 6 | H, HZ, M, RZ, R, Z | 8 | HRZE, RMZE, BPaL, BPa, RM, BL, PaL, B | -2 | - |
| **RZ** | 10 | 5 | H, HZ, M, R, Z | 9 | HRZE, RMZE, BPaL, BPa, RM, BL, Pa, PaL, B | -4 | - |
| **HZ** | 11 | 3 | H, R, Z | 10 | HRZE, RMZE, BPaL, BPa, RZ, RM, BL, Pa, PaL, B | -7 | M |
| **M** | 11 | 3 | H, R, Z | 10 | HRZE, RMZE, BPaL, BPa, RZ, RM, BL, Pa, PaL, B | -7 | HZ |
| **Z** | 13 | 2 | H, R | 12 | HRZE, RMZE, BPaL, BPa, HZ, M, RZ, RM, BL, Pa, PaL, B | -10 | - |
| **R** | 14 | 1 | H | 13 | HRZE, RMZE, BPaL, BPa, HZ, M, RZ, RM, BL, Z, Pa, PaL, B | -12 | - |
| **H** | 15 | 0 | - | 14 | HRZE, RMZE, BPaL, BPa, HZ, M, RZ, R, RM, BL, Z, Pa, PaL, B | -14 | - |

Table S13: **Scores and ranks of each regimen in the regimen set from *GranSim* studies, considering high-CFU granulomas.** Dark purple-shaded regimens are candidates for the most potent regimens and regimens with the white background are candidates for the least potent regimens. The regimens in the light purple-shaded area are those regimens for which AUSCs are higher (and lower) than at least one regimen in the regimen set.

| **Reference regimen** | **Rank** | **Nr of regimens significantly worse than the reference regimen (w)** | **Regimens that are significantly worse than the reference regimen** | **Nr of regimens significantly better than the reference regimen (b)** | **Regimens that are significantly better than the reference regimen** | **Score (w-b)** | **Reference regimen is not significantly different than** |
| --- | --- | --- | --- | --- | --- | --- | --- |
| **RMZE** | 1 | 12 | HRZE, H, HZ, M, RZ, R, RM, BL, Z, Pa, PaL, B | 0 | - | 12 | BPaL, BPa |
| **BPaL** | 1 | 12 | HRZE, H, HZ, M, RZ, R, RM, BL, Z, Pa, PaL, B | 0 | - | 12 | RMZE, BPa |
| **BPa** | 1 | 12 | HRZE, H, HZ, M, RZ, R, RM, BL, Z, Pa, PaL, B | 0 | - | 12 | RMZE, BPaL |
| **BL** | 4 | 11 | HRZE, H, HZ, M, RZ, R, RM, Z, Pa, PaL, B | 3 | RMZE, BPaL, BPa | 8 | - |
| **RM** | 5 | 10 | HRZE, H, HZ, M, RZ, R, Z, Pa, PaL, B | 4 | RMZE, BPaL, BPa, BL | 6 | - |
| **HRZE** | 6 | 8 | H, HZ, M, RZ, R, Z, Pa, PaL | 5 | RMZE, BPaL, BPa, RM, BL | 3 | B |
| **B** | 7 | 7 | H, HZ, M, RZ, R, Z, Pa | 5 | RMZE, BPaL, BPa, RM, BL | 2 | HRZE, PaL |
| **PaL** | 8 | 6 | H, HZ, M, RZ, R, Z | 6 | HRZE, RMZE, BPaL, BPa, RM, BL | 0 | Pa, B |
| **Pa** | 9 | 6 | H, HZ, M, RZ, R, Z | 7 | HRZE, RMZE, BPaL, BPa, RM, BL, B | -1 | PaL |
| **RZ** | 10 | 5 | H, HZ, M, R, Z | 9 | HRZE, RMZE, BPaL, BPa, RM, BL, Pa, PaL, B | -4 | - |
| **HZ** | 11 | 4 | H, M, R, Z | 10 | HRZE, RMZE, BPaL, BPa, RZ, RM, BL, Pa, PaL, B | -6 | - |
| **M** | 12 | 3 | H, R, Z | 11 | HRZE, RMZE, BPaL, BPa, HZ, RZ, RM, BL, Pa, PaL, B | -8 | - |
| **R** | 13 | 1 | H | 12 | HRZE, RMZE, BPaL, BPa, HZ, M, RZ, RM, BL, Pa, PaL, B | -11 | Z |
| **Z** | 14 | 0 | - | 12 | HRZE, RMZE, BPaL, BPa, HZ, M, RZ, RM, BL, Pa, PaL, B | -12 | H, R |
| **H** | 15 | 0 | - | 13 | HRZE, RMZE, BPaL, BPa, HZ, M, RZ, R, RM, BL, Pa, PaL, B | -13 | Z |

Table S14: **Scores and ranks of each regimen in the regimen set from *GranSim* studies, considering low-CFU granulomas.** Dark purple-shaded regimens are candidates for the most potent regimens and regimens with the white background are candidates for the least potent regimens. The regimens in the light purple-shaded area are those regimens for which AUSCs are higher (and lower) than at least one regimen in the regimen set.

| **Reference regimen** | **Rank** | **Nr of regimens significantly worse than the reference regimen (w)** | **Regimens that are significantly worse than the reference regimen** | **Nr of regimens significantly better than the reference regimen (b)** | **Regimens that are significantly better than the reference regimen** | **Score (w-b)** | **Reference regimen is not significantly different than** |
| --- | --- | --- | --- | --- | --- | --- | --- |
| **RMZE** | 1 | 14 | HRZE, BPaL, BPa, H, HZ, M, RZ, R, RM, BL, Z, Pa, PaL, B | 0 |  | 14 |  |
| **RM** | 2 | 13 | HRZE, BPaL, BPa, H, HZ, M, RZ, R, BL, Z, Pa, PaL, B | 1 | RMZE | 12 |  |
| **PaL** | 3 | 9 | HRZE, H, HZ, RZ, R, BL, Z, Pa, B | 2 | RMZE, RM | 7 | BPaL, BPa, M |
| **BPaL** | 4 | 8 | HRZE, H, HZ, RZ, R, BL, Z, B | 2 | RMZE, RM | 6 | BPa, M, Pa, PaL |
| **BPa** | 4 | 8 | HRZE, H, HZ, RZ, R, BL, Z, B | 2 | RMZE, RM | 6 | BPaL, M, Pa, PaL |
| **M** | 4 | 8 | HRZE, H, HZ, RZ, R, BL, Z, B | 2 | RMZE, RM | 6 | BPaL, BPa, Pa, PaL |
| **Pa** | 7 | 8 | HRZE, H, HZ, RZ, R, BL, Z, B | 3 | RMZE, RM, PaL | 5 | BPaL, BPa, M |
| **BL** | 8 | 7 | HRZE, H, HZ, RZ, R, Z, B | 7 | RMZE, BPaL, BPa, M, RM, Pa, PaL | 0 | - |
| **B** | 9 | 6 | HRZE, H, HZ, RZ, R, Z | 8 | RMZE, BPaL, BPa, M, RM, BL, Pa, PaL | -2 | - |
| **HRZE** | 10 | 5 | H, HZ, RZ, R, Z | 9 | RMZE, BPaL, BPa, M, RM, BL, Pa, PaL, B | -4 | - |
| **RZ** | 11 | 4 | H, HZ, R, Z | 10 | HRZE, RMZE, BPaL, BPa, M, RM, BL, Pa, PaL, B | -6 | - |
| **HZ** | 12 | 3 | H, R, Z | 11 | HRZE, RMZE, BPaL, BPa, M, RZ, RM, BL, Pa, PaL, B | -8 | - |
| **Z** | 13 | 2 | H, R | 12 | HRZE, RMZE, BPaL, BPa, HZ, M, RZ, RM, BL, Pa, PaL, B | -10 | - |
| **R** | 14 | 1 | H | 13 | HRZE, RMZE, BPaL, BPa, HZ, M, RZ, RM, BL, Z, Pa, PaL, B | -12 | - |
| **H** | 15 | 0 | - | 14 | HRZE, RMZE, BPaL, BPa, HZ, M, RZ, R, RM, BL, Z, Pa, PaL, B | -14 | - |

**References**

1. Segovia-Juarez JL, Ganguli S, Kirschner D. Identifying control mechanisms of granuloma formation during M. tuberculosis infection using an agent-based model. J Theor Biol. 2004;231(3):357-76. doi: 10.1016/j.jtbi.2004.06.031. PubMed PMID: 15501468.

2. Ray JC, Flynn JL, Kirschner DE. Synergy between individual TNF-dependent functions determines granuloma performance for controlling Mycobacterium tuberculosis infection. J Immunol. 2009;182(6):3706-17. doi: 10.4049/jimmunol.0802297. PubMed PMID: 19265149; PubMed Central PMCID: PMCPMC3182770.

3. Global tuberculosis report 2022. Geneva: World Health Organization; 2022.

4. Pienaar E, Linderman JJ, Kirschner DE. Emergence and selection of isoniazid and rifampin resistance in tuberculosis granulomas. PLoS One. 2018;13(5):e0196322. Epub 20180510. doi: 10.1371/journal.pone.0196322. PubMed PMID: 29746491; PubMed Central PMCID: PMCPMC5944939.

5. Pienaar E, Cilfone NA, Lin PL, Dartois V, Mattila JT, Butler JR, et al. A computational tool integrating host immunity with antibiotic dynamics to study tuberculosis treatment. J Theor Biol. 2015;367:166-79. Epub 20141209. doi: 10.1016/j.jtbi.2014.11.021. PubMed PMID: 25497475; PubMed Central PMCID: PMCPMC4332617.

6. Pienaar E, Dartois V, Linderman JJ, Kirschner DE. In silico evaluation and exploration of antibiotic tuberculosis treatment regimens. BMC Syst Biol. 2015;9:79. Epub 20151114. doi: 10.1186/s12918-015-0221-8. PubMed PMID: 26578235; PubMed Central PMCID: PMCPMC4650854.

7. Pienaar E, Sarathy J, Prideaux B, Dietzold J, Dartois V, Kirschner DE, et al. Comparing efficacies of moxifloxacin, levofloxacin and gatifloxacin in tuberculosis granulomas using a multi-scale systems pharmacology approach. PLoS Comput Biol. 2017;13(8):e1005650. Epub 20170817. doi: 10.1371/journal.pcbi.1005650. PubMed PMID: 28817561; PubMed Central PMCID: PMCPMC5560534.

8. Cicchese JM, Sambarey A, Kirschner D, Linderman JJ, Chandrasekaran S. A multi-scale pipeline linking drug transcriptomics with pharmacokinetics predicts in vivo interactions of tuberculosis drugs. Sci Rep. 2021;11(1):5643. Epub 20210311. doi: 10.1038/s41598-021-84827-0. PubMed PMID: 33707554; PubMed Central PMCID: PMCPMC7971003.

9. Budak M, Cicchese JM, Maiello P, Borish HJ, White AG, Chishti HB, et al. Optimizing tuberculosis treatment efficacy: Comparing the standard regimen with Moxifloxacin-containing regimens. PLoS Comput Biol. 2023;19(6):e1010823. Epub 20230615. doi: 10.1371/journal.pcbi.1010823. PubMed PMID: 37319311; PubMed Central PMCID: PMCPMC10306236.

10. Strydom N, Gupta SV, Fox WS, Via LE, Bang H, Lee M, et al. Tuberculosis drugs' distribution and emergence of resistance in patient's lung lesions: A mechanistic model and tool for regimen and dose optimization. PLoS Med. 2019;16(4):e1002773. Epub 20190402. doi: 10.1371/journal.pmed.1002773. PubMed PMID: 30939136; PubMed Central PMCID: PMCPMC6445413.

11. Dhillon J, Andries K, Phillips PP, Mitchison DA. Bactericidal activity of the diarylquinoline TMC207 against Mycobacterium tuberculosis outside and within cells. Tuberculosis (Edinb). 2010;90(5):301-5. Epub 20100821. doi: 10.1016/j.tube.2010.07.004. PubMed PMID: 20732832.

12. Sarathy JP, Via LE, Weiner D, Blanc L, Boshoff H, Eugenin EA, et al. Extreme Drug Tolerance of Mycobacterium tuberculosis in Caseum. Antimicrob Agents Chemother. 2018;62(2). Epub 20180125. doi: 10.1128/AAC.02266-17. PubMed PMID: 29203492; PubMed Central PMCID: PMCPMC5786764.

13. Sarathy JP, Xie M, Jones RM, Chang A, Osiecki P, Weiner D, et al. A Novel Tool to Identify Bactericidal Compounds against Vulnerable Targets in Drug-Tolerant M. tuberculosis found in Caseum. mBio. 2023;14(2):e0059823. Epub 20230405. doi: 10.1128/mbio.00598-23. PubMed PMID: 37017524; PubMed Central PMCID: PMCPMC10127596.

14. de Miranda Silva C, Hajihosseini A, Myrick J, Nole J, Louie A, Schmidt S, et al. Effect of Moxifloxacin plus Pretomanid against. Antimicrob Agents Chemother. 2019;63(1). Epub 20181221. doi: 10.1128/AAC.01695-18. PubMed PMID: 30397058; PubMed Central PMCID: PMCPMC6325209.

15. Larkins-Ford J, Greenstein T, Van N, Degefu YN, Olson MC, Sokolov A, et al. Systematic measurement of combination-drug landscapes to predict in vivo treatment outcomes for tuberculosis. Cell Syst. 2021;12(11):1046-63.e7. Epub 20210831. doi: 10.1016/j.cels.2021.08.004. PubMed PMID: 34469743; PubMed Central PMCID: PMCPMC8617591.

16. de Knegt GJ, van der Meijden A, de Vogel CP, Aarnoutse RE, de Steenwinkel JE. Activity of moxifloxacin and linezolid against Mycobacterium tuberculosis in combination with potentiator drugs verapamil, timcodar, colistin and SQ109. Int J Antimicrob Agents. 2017;49(3):302-7. Epub 20170202. doi: 10.1016/j.ijantimicag.2016.11.027. PubMed PMID: 28162983.

17. Chandrasekaran S, Cokol-Cakmak M, Sahin N, Yilancioglu K, Kazan H, Collins JJ, et al. Chemogenomics and orthology-based design of antibiotic combination therapies. Mol Syst Biol. 2016;12(5):872. Epub 20160524. doi: 10.15252/msb.20156777. PubMed PMID: 27222539; PubMed Central PMCID: PMCPMC5289223.

18. Ma S, Jaipalli S, Larkins-Ford J, Lohmiller J, Aldridge BB, Sherman DR, et al. Transcriptomic Signatures Predict Regulators of Drug Synergy and Clinical Regimen Efficacy against Tuberculosis. mBio. 2019;10(6). Epub 20191112. doi: 10.1128/mBio.02627-19. PubMed PMID: 31719182; PubMed Central PMCID: PMCPMC6851285.

19. Marino S, Hogue IB, Ray CJ, Kirschner DE. A methodology for performing global uncertainty and sensitivity analysis in systems biology. J Theor Biol. 2008;254(1):178-96. Epub 20080420. doi: 10.1016/j.jtbi.2008.04.011. PubMed PMID: 18572196; PubMed Central PMCID: PMCPMC2570191.

20. Hult C, Mattila JT, Gideon HP, Linderman JJ, Kirschner DE. Neutrophil Dynamics Affect. Front Immunol. 2021;12:712457. Epub 20211005. doi: 10.3389/fimmu.2021.712457. PubMed PMID: 34675916; PubMed Central PMCID: PMCPMC8525425.

21. Joslyn LR, Linderman JJ, Kirschner DE. A virtual host model of Mycobacterium tuberculosis infection identifies early immune events as predictive of infection outcomes. J Theor Biol. 2022;539:111042. Epub 20220131. doi: 10.1016/j.jtbi.2022.111042. PubMed PMID: 35114195; PubMed Central PMCID: PMCPMC9169921.

22. Renardy M, Joslyn LR, Millar JA, Kirschner DE. To Sobol or not to Sobol? The effects of sampling schemes in systems biology applications. Math Biosci. 2021;337:108593. Epub 20210416. doi: 10.1016/j.mbs.2021.108593. PubMed PMID: 33865847; PubMed Central PMCID: PMCPMC8184610.

23. Lin PL, Maiello P, Gideon HP, Coleman MT, Cadena AM, Rodgers MA, et al. PET CT Identifies Reactivation Risk in Cynomolgus Macaques with Latent M. tuberculosis. PLoS Pathog. 2016;12(7):e1005739. Epub 20160705. doi: 10.1371/journal.ppat.1005739. PubMed PMID: 27379816; PubMed Central PMCID: PMCPMC4933353.

24. Via LE, England K, Weiner DM, Schimel D, Zimmerman MD, Dayao E, et al. A sterilizing tuberculosis treatment regimen is associated with faster clearance of bacteria in cavitary lesions in marmosets. Antimicrob Agents Chemother. 2015;59(7):4181-9. Epub 20150504. doi: 10.1128/AAC.00115-15. PubMed PMID: 25941223; PubMed Central PMCID: PMCPMC4468655.

25. Via LE, Weiner DM, Schimel D, Lin PL, Dayao E, Tankersley SL, et al. Differential virulence and disease progression following Mycobacterium tuberculosis complex infection of the common marmoset (Callithrix jacchus). Infect Immun. 2013;81(8):2909-19. Epub 20130528. doi: 10.1128/IAI.00632-13. PubMed PMID: 23716617; PubMed Central PMCID: PMCPMC3719573.

26. Beites T, O'Brien K, Tiwari D, Engelhart CA, Walters S, Andrews J, et al. Plasticity of the Mycobacterium tuberculosis respiratory chain and its impact on tuberculosis drug development. Nat Commun. 2019;10(1):4970. Epub 20191031. doi: 10.1038/s41467-019-12956-2. PubMed PMID: 31672993; PubMed Central PMCID: PMCPMC6823465.

27. Diacon AH, De Jager VR, Dawson R, Narunsky K, Vanker N, Burger DA, et al. Fourteen-Day Bactericidal Activity, Safety, and Pharmacokinetics of Linezolid in Adults with Drug-Sensitive Pulmonary Tuberculosis. Antimicrob Agents Chemother. 2020;64(4). Epub 20200324. doi: 10.1128/AAC.02012-19. PubMed PMID: 31988102; PubMed Central PMCID: PMCPMC7179319.

28. Li M, Saviolakis GA, El-Amin W, Makhene MK, Osborn B, Nedelman J, et al. Phase 1 Study of the Effects of the Tuberculosis Treatment Pretomanid, Alone and in Combination With Moxifloxacin, on the QTc Interval in Healthy Volunteers. Clin Pharmacol Drug Dev. 2021;10(6):634-46. Epub 20201230. doi: 10.1002/cpdd.898. PubMed PMID: 33378139; PubMed Central PMCID: PMCPMC8246780.

29. Shao G, Bao Z, Davies Forsman L, Paues J, Werngren J, Niward K, et al. Population pharmacokinetics and model-based dosing evaluation of bedaquiline in multidrug-resistant tuberculosis patients. Front Pharmacol. 2023;14:1022090. Epub 20230327. doi: 10.3389/fphar.2023.1022090. PubMed PMID: 37050904; PubMed Central PMCID: PMCPMC10083270.

30. Diacon AH, Pym A, Grobusch M, Patientia R, Rustomjee R, Page-Shipp L, et al. The diarylquinoline TMC207 for multidrug-resistant tuberculosis. N Engl J Med. 2009;360(23):2397-405. doi: 10.1056/NEJMoa0808427. PubMed PMID: 19494215.

31. Zvada SP, Denti P, Sirgel FA, Chigutsa E, Hatherill M, Charalambous S, et al. Moxifloxacin population pharmacokinetics and model-based comparison of efficacy between moxifloxacin and ofloxacin in African patients. Antimicrob Agents Chemother. 2014;58(1):503-10. Epub 20131104. doi: 10.1128/AAC.01478-13. PubMed PMID: 24189253; PubMed Central PMCID: PMCPMC3910772.
